# Effluent and serum protein *N*-glycosylation is associated with inflammation and peritoneal membrane transport characteristics in peritoneal dialysis patients

**DOI:** 10.1038/s41598-018-19147-x

**Published:** 2018-01-17

**Authors:** Evelina Ferrantelli, Karima Farhat, Agnes L. Hipgrave Ederveen, Karli R. Reiding, Robert H. J. Beelen, Frans J. van Ittersum, Manfred Wuhrer, Viktoria Dotz

**Affiliations:** 1 VU University Medical Center, Department of Molecular Cell Biology and Immunology, Amsterdam, The Netherlands; 2 VU University Medical Center, Department of Nephrology, Amsterdam, The Netherlands; 3 Center for Proteomics and Metabolomics, Leiden University Medical Center, Leiden, The Netherlands; 4 VU University Amsterdam, Division of BioAnalytical Chemistry, Amsterdam, The Netherlands; 5 Present Address: Academic Medical Center, Tytgat Institute for Gastrointestinal and Liver Disease, Amsterdam, The Netherlands

## Abstract

Mass spectrometric glycomics was used as an innovative approach to identify biomarkers in serum and dialysate samples from peritoneal dialysis (PD) patients. PD is a life-saving treatment worldwide applied in more than 100,000 patients suffering from chronic kidney disease. PD treatment uses the peritoneum as a natural membrane to exchange waste products from blood to a glucose-based solution. Daily exposure of the peritoneal membrane to these solutions may cause complications such as peritonitis, fibrosis and inflammation which, in the long term, lead to the failure of the treatment. It has been shown in the last years that protein *N-*glycosylation is related to inflammatory and fibrotic processes. Here, by using a recently developed MALDI-TOF-MS method with linkage-specific sialic acid derivatisation, we showed that alpha2,6-sialylation, especially in triantennary *N-*glycans from peritoneal effluents, is associated with critical clinical outcomes in a prospective cohort of 94 PD patients. Moreover, we found an association between the levels of presumably immunoglobulin-G-related glycans as well as galactosylation of diantennary glycans with PD-related complications such as peritonitis and loss of peritoneal mesothelial cell mass. The observed glycomic changes point to changes in protein abundance and protein-specific glycosylation, representing candidate functional biomarkers of PD and associated complications.

## Introduction

Peritoneal dialysis (PD) is a safe treatment modality for patients with end-stage kidney disease. PD uses a patient’s peritoneum as a natural membrane to remove waste products and fluid from the blood to the dialysis solution by using mainly glucose as osmotic agent. Currently, PD is used by >100,000 end-stage renal disease patients worldwide and accounts for approximately 11% of the dialysis population^1^. However, long term exposure to PD solutions is associated with functional and structural alterations of the peritoneum which include neovascularisation, thickening of the peritoneal membrane, peritonitis^2–4^ as well as epithelial-to-mesenchymal transition (EMT). The latter is known as a process whereby the mesothelial cells lining the peritoneal cavity lose their epithelial characteristics to acquire a fibroblast-like phenotype^5,6^. Taken together all these events lead to ultrafiltration failure resulting in critical clinical outcomes^7^.

Besides acting as a biological barrier, the peritoneum, and more specifically its mesothelial cell monolayer, also functions as a secretory organ which synthesizes and secretes cytokines responsible for the regulation of peritoneal permeability and local host defence. During EMT, the secretion of pro-inflammatory factors^8^, such as transforming growth factor-β1 (TGFβ-1) increases significantly. Besides inducing EMT^9^, TGFβ-1 has been shown to promote peritoneal fibrosis via various pro-fibrotic events including proliferation of fibroblasts and extracellular matrix deposition^10^. TGFβ-1-induced EMT was shown to affect cellular protein glycosylation in normal mouse mammary gland epithelial cells^11^ as well as cancer cells^12^. Loss of mesothelial cells is also represented by a decrease in the levels of cancer antigen 125 (CA-125), a marker of peritoneal cell mass and function^13^, while hyaluronic acid (HA) deposition is characteristic of peritoneal fibrosis subsequent to dialysis treatment^14^. Fibrosis and angiogenesis seem to occur together in the peritoneal tissues^15^. Consequently, an increase in TGFβ-1 levels is often associated with high levels of vascular endothelial growth factor (VEGF), which is known to stimulate angiogenesis via capillary tube formation^16^.

Activated mesothelial cells also produce chemotactic cytokines leading to the recruitment of leukocytes and rapid accumulation of neutrophils, later replaced by monocytes and/or macrophages and lymphocytes, into the peritoneum. This scenario is typical of a peritonitis episode, a common PD-related event driven by cytokines such as interleukin (IL)-6, IL-8, monocytes chemotactic protein 1 (MCP-1) and many others^17^.

In clinical practice, effluent markers such as IL-6 and CA-125 are used to assess peritoneal functionality and morphology^18–20^, but their role as predictors for peritoneal membrane failure is still questioned. Changes in the levels of cytokines detected in peritoneal effluents collected from patients indeed reflect the peritoneal morphological changes, yet only at a very late stage^21^. Thus, relevant predictors for PD technique failure at an earlier stage are still needed.

Protein losses have been considered a major drawback in PD, and have recently been investigated in a few PD proteomics studies. Losses of protein in PD effluent (PDE) range from 5 to 15 g/day, depending on various factors, such as a patient’s clinical status or PD fluid composition^22^. When compared to the overall human proteome, some quantitative differences in PDE have been shown, especially regarding immune-related and vitamin-binding proteins, coagulation factors and apolipoproteins that are known to be locally produced by the peritoneum^23,24^. Differences in the PD proteome have been shown in patients in relation to, *e.g*., diabetes^25^, or different peritoneal transport characteristics^25,26^. However, proteomic studies are scarce and have mainly been conducted in small cohorts.

Moreover, little is known about PD-related protein glycosylation, which is a post-translational modification of influence to protein functions such as cell adhesion, signal transduction, receptor activation, molecular trafficking and systemic clearance^27^. Protein *N-*glycosylation is known to be related to inflammatory^28^ and fibrotic^29^ processes. Previously, we reported on early *N-*glycosylation changes in mice with zymosan-induced peritonitis by using linkage-specific derivatisation of sialic acids^30^.

In the present study we adapted this glycomic methodology to clinical PDE samples that are challenging for glycan analysis due to lower protein concentrations as compared to serum and due to the presence of interfering compounds, i.e. hexose polymers. Thus, we present a novel and attractive approach for biomarker identification and demonstrate that changes in the glycosylation profile are associated with PD-related complications such as peritonitis, inflammation and mesothelial cell loss in a prospective cohort of 94 PD patients.

## Results and Discussion

### Study design and glycomic profiling

Serum and effluent samples were collected from PD patients in 6-months intervals for up to 24 months (Table 1). Clinical parameters, such as cytokine levels, peritoneal functionality or adverse events were furthermore assessed (Table 2)^31^. To increase statistical power, data was not analysed per treatment group (Dianeal or Physioneal), but treatment group was included as a covariate in the regression models.

**Table 1:** Patient numbers and age at baseline and follow-up.

Study month	Number of patients		Age in years (mean ± SD)	
	M	F	M	F
0	63	31	62.05 ± 12.47	59.77 ± 16.04
1–6	49	28	62.70 ± 12.66	60.35 ± 16.28
7–12	43	23	63.33 ± 12.98	58.77 ± 16.70
13–18	27	14	63.79 ± 13.15	58.82 ± 14.25
19–24	20	11	64.00 ± 13.96	58.29 ± 15.46

Given are the numbers of patients (M, male/F, female) left at the respective time periods of sample collection for glycomic profiling and/or determination of clinical parameters as described in the experimental section.

**Table 2:** Clinical characteristics at baseline.

Clinical parameter	Number of patients		Median ± interquartile range	
	M	F	M	F
Diabetes	10	22		
CAPD/APD	53/10	27/4		
Dianeal/Physioneal	55/8	23/8		
Time on PD (months)	62	31	16.80 ± 20.50	12.20 ± 21.50
DPCrea4	63	31	0.74 ± 0.14	0.70 ± 0.15
Ultrafiltration_PET (mL)	63	31	400.00 ± 344.30	540.00 ± 398.20
IL-6 (ρg/mL)	61	30	96.74 ± 138.87	71.16 ± 119.13
IL-8 (ρg/mL)	60	30	35.44 ± 93.00	19.81 ± 49.25
MCP-1 (ρg/mL)	61	30	247.01 ± 178.99	171.91 ± 118.38
TGFβ-1 (ρg/mL)	61	30	75.05 ± 82.78	93.43 ± 77.05
VEGF (ρg/mL)	60	29	146.94 ± 114.11	102.58 ± 140.27
CA-125 (ρg/mL)	61	30	19.60 ± 18.40	21.45 ± 13.6
HA (ρg/mL)	61	30	179.11 ± 138.27	148.52 ± 129.13

Given are the numbers of patients (M, male/F, female) with available data on the stated parameter. See Table 1 for total patient number at study month 0. The following clinical parameters were used as confounders for regression analysis with GEE, next to age and sex: Diabetes; CAPD, Continuous Ambulatory Peritoneal Dialysis; APD, Automated Peritoneal Dialysis; Dianeal or Physioneal treatment group; time on PD (refers to the total time in months after a patient was first introduced to PD treatment). The Dianeal group was randomised into two treatment groups at baseline, i.e. 27/11 male/female patients continued with Dianeal for 24 months, while 28/12 male/female patients switched to Physioneal for the following 24 months. DPCrea4 is the ratio of creatinine in the effluent vs. plasma during 4-h dwell time.

**Figure 1:**
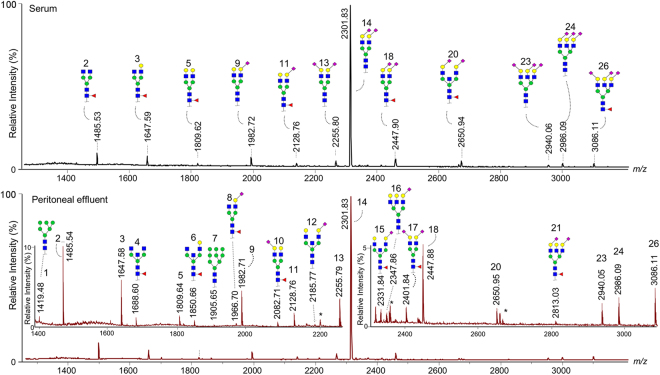
MALDI-TOF mass spectra of a patient’s serum (upper) and peritoneal effluent (lower) protein-derived *N*-glycans. After enzymatic release of glycans, sialic acids were stabilised in linkage-specific manner and analysed by positive-ion reflectron mode MALDI-TOF-MS. Blue square, *N*-acetylglucosamine; yellow circle, galactose; green circle, mannose; red triangle, fucose; purple diamond oriented to the right, α2,6-linked *N*-acetylneuraminic acid; purple diamond oriented to the left, α2,3-linked *N*-acetylneuraminic acid; asterisks indicate peaks of non-*N*-glycan origin. Structures are proposed for the 12 most abundant species in the upper mass spectrum, while the lower panel contains 11 additional structures in the two zoomed areas, i.e. peaks 1, 4, 6–8, 10, 12, 15–17, and 21. For the complete list of, in total, 26 detected glycan species, see Supporting Information Table S-1.

The *N-*glycomic profiles of the patients’ serum and PDE samples were acquired by MALDI-TOF-MS analysis after protein immobilisation, enzymatic *N-*glycan release, and sialic acid stabilisation with differentiation of α2,3- and α2,6-linked sialic acids^32^. Importantly, the immobilisation of proteins on PVDF membrane was necessary for freeing PDE samples from hexose polymers that originate from PD fluid and that would otherwise interfere with *N-*glycomic analysis (Supporting Information Fig. S-1). To ensure unambiguous mass assignment, we excluded 15 detected compositions from further analysis due to overlapping masses of *N*-glycans with possible residual dextran interferences in the effluent. Structural features were then assigned to the remaining 26 *N-*glycan compositions in serum and effluent each (Fig. 1, Supporting Information Table S-1), in accordance with the established knowledge of the human plasma *N-*glycome and its biosynthetic pathways^32–34^. Twelve derived traits were calculated representing major structural glycomic features, i.e. complexity (high-mannosidic, di- and tri-antennary species), bisection, galactosylation, fucosylation, α2,3- and α2,6-sialylation, as well as the sum of presumably IgG-related glycans (Supporting Information Table S-2). No tetraantennary glycans were detected, which was attributed to the rather limited sensitivity of the workflow, most probably caused by the limited capacity of the PVDF membrane used for protein immobilisation. As a comparison, when using our in-solution glycan release, we are able to detect more than 90 different species in human plasma and murine peritoneal effluent^30,32^. However, in the current study we chose to apply the same membrane-based sample method and data processing criteria to both effluent and serum, to ensure the comparability of the glycomes from the two biofluids and assess the usefulness of effluent samples for biomarker discovery.

### Effluent versus serum protein glycosylation

**Figure 2:**
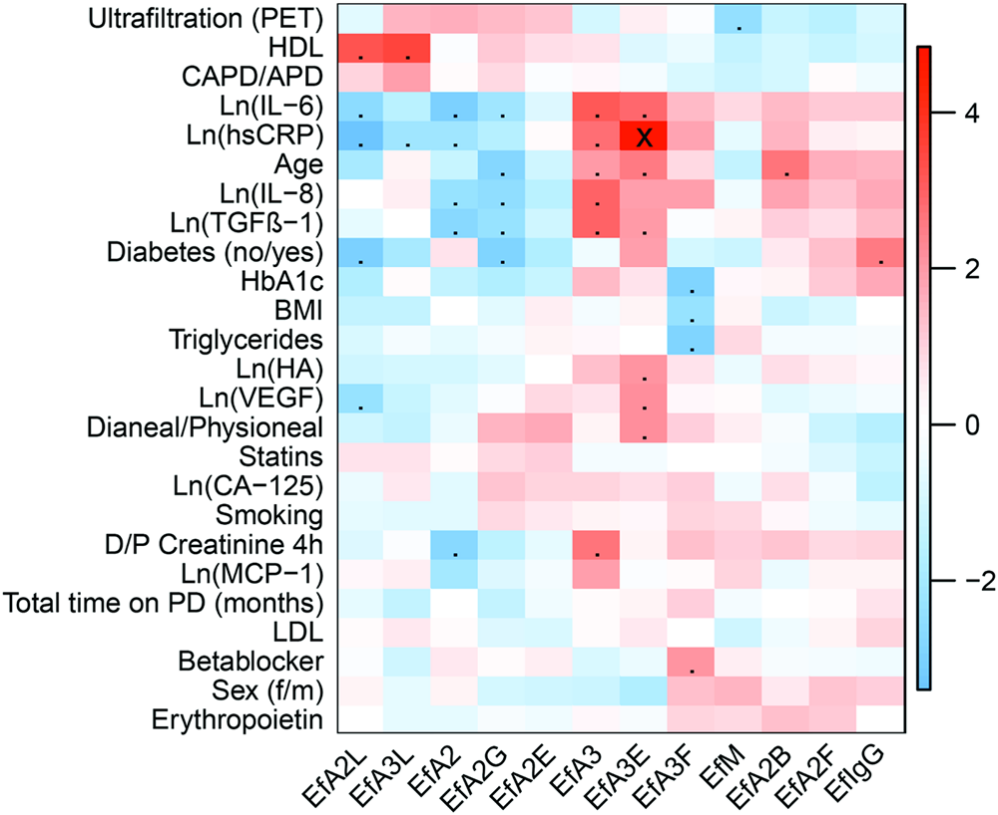
Heatmap resulting from the t- or Wald statistics (β/SE) of the associations between clinical parameters and glycans from PD effluent at baseline. The ranking of the categorical variables is matching the listing order of their categories, e.g. for Sex, female = 0, male = 1; for the binary variables, i.e. Diabetes, Statins, Smoking, Betablocker, and Erythropoietin the coding was no = 0 and yes = 1. Dots refer to p < 0.05, while crosses represent p-values below the significance threshold corrected for multiple testing by the Bonferroni method (α = 0.00017). Colour codes indicate t- or Wald statistics as depicted in the colour scale to the right of the heat map. Hierarchical clustering (Euclidian distance, complete linkage) was applied for the sorting of the heatmap variables.

This is the first study showing associations of the total protein *N*-glycomes in serum and effluent with important clinical parameters in PD. Associations between plasma *N*-glycans and various metabolic and inflammatory parameters were recently described in a large cohort of healthy individuals^35^. Likewise, we found associations between *N*-glycan features in serum as well as effluent from PD patients with inflammatory markers, such as IL-6 and high-sensitivity C-reactive protein (hsCRP), and, moreover, with PD-related parameters such as D/P creatinine after 4 hours (PET) and TGFβ-1 (Fig. 2, Supporting Information Fig. S-2A).

Spearman correlation analysis of the 26 glycan species detected in 91 serum and 87 PDE samples at baseline revealed several discrepancies between the two *N-*glycomic profiles, recognisable in a correlation coefficient of the same detected species of lower than +1 (Supporting Information Fig. S-1B). This becomes specifically apparent for the glycans H4N4E1F1, H5N4L1F1, H6N5L1E1, and H6N5E2F1 (peaks 8, 10, 19, and 21). In contrast, fucosylated, non-sialylated diantennary glycans – both with and without bisecting GlcNAc – correlated well between serum and effluent (peaks 2–6 in Fig. 1 and Supporting Information Table S-1). As for serum, one may assume that these glycans may be largely IgG-derived^36^. Although we used the same laboratory procedures to prepare and analyse serum and PDE, technical variation may contribute to some of the observed glycomic discrepancies between the two sample types. Nevertheless, we expect differences to largely arise from biological sources.

When assigning the 26 detected species to 12 derived traits, correlations between serum and PDE glycans increased (Supporting Information Fig. S-2C). This is in line with previous reports indicating a higher technical robustness and often also biological relevance of derived traits in glycomic comparisons as compared to direct traits^37,38^.

**Figure 3:**
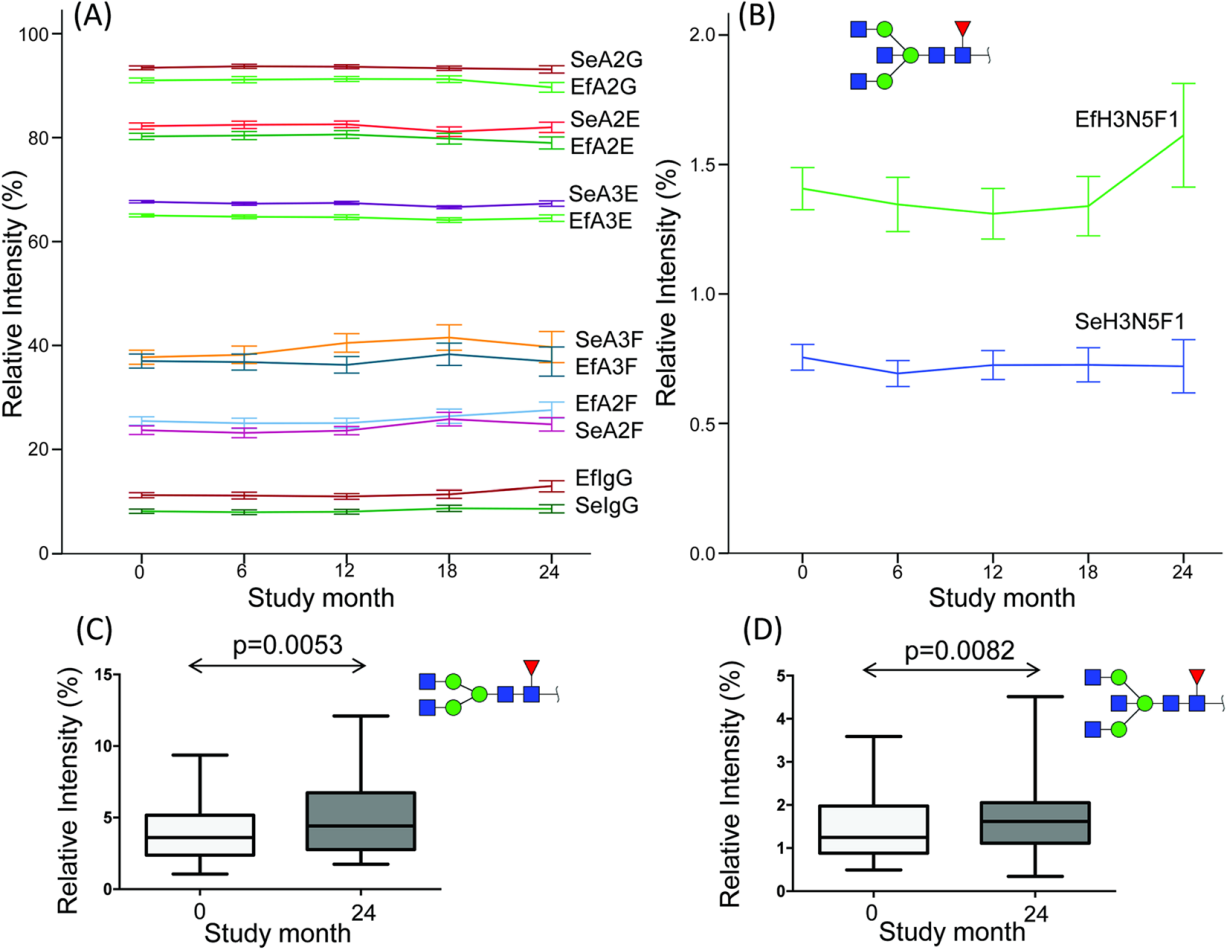
Selected *N*-glycan traits from serum (Se) and effluent (Ef) over time. (**A** and **B**) Mean ± SE values of all patients over time classes as defined in Table 1. (**A**) At baseline (study month 0) all p-values were below the Bonferroni-adjusted significance threshold α = 0.0042 in paired Wilcoxon signed-rank tests for serum vs. effluent for A2G, A2E, A3E, A2F, IgG-related traits (N = 85 each); A3F was not significantly different at baseline. (**C** and **D**) Min/Max-values and boxplots with p-values from a paired Wilcoxon signed-rank test (two-tailed) for IgG-related direct traits in effluent samples from 24 patients at baseline vs. 24 months.

At baseline, differences between serum and PDE glycosylation levels for the derived traits M, A2, A3, A2F, A2G, A2E, A3E, and IgG were observed in paired Wilcoxon signed-rank tests, whereas no differences could be found in A3F, bisection, and α2,3-sialylation (Fig. 3A, Supporting Information Fig. S-3A-C). Similar differences were observed during the 24 months follow-up as is discussed in more detail in the following section. Interestingly, in a recently published study involving healthy mice, high-mannose glycans were reported to be more abundant in PDE than in plasma^30^, whereas our data in PD patients shows the reverse (Supporting Information Fig. S-3A). Moreover, the relative α2,6-sialylation of both di- and triantennary glycans (A2E and A3E) was higher in serum than in PDE (Fig. 3A), while in healthy mice we did not observe differences in overall or linkage-specific sialylation between plasma and peritoneal effluent *N-*glycans^30^.

### N-glycan features are associated with inflammatory markers and PD-related parameters in a longitudinal setting

In literature, the total protein concentration in PDE is reported to remain stable over several months up to years on PD treatment^22,39^. Data on PDE protein composition over time are scarce and proteomic changes, if any, are reported in studies with low statistical power^40^. However, pathological and morphological changes of the peritoneum can occur with duration of PD treatment and may be reflected by the effluent composition^18^. Accordingly, a few trends could be observed in our glycomics data, in particular, in the effluent towards the end of the study. For instance, the galactosylation of diantennary glycans (A2G) showed a downward trend at 24-months follow-up in the PDE, but not in serum (Fig. 3A, Supporting Information Fig. S-3D). This could be mainly attributed to an increase of the agalactosylated glycan compositions H3N4F1 and H3N5F1 (Fig. 3B-D), since the relative abundance of the mono- and digalactosylated – presumably IgG-related – species remained stable over time in PDE (Supporting Information Fig. S-3D). A lower galactosylation of IgG is related to a pro-inflammatory state in different pathological conditions^41,42^.

Assessing the dynamic changes in glycosylation in the three treatment groups separately did not retrieve significant results, most likely due to a lack of statistical power (N = 9, 12, and 3 effluent glycome data pairs for baseline *vs*. 24 months in group 1, 2, and 3, respectively). However, group 2 (switched from Dianeal to Physioneal) did show several interesting trends (Supporting Information Fig. S-3E). For example, effluent IgG glycans tended to increase, while the triantennary glycan H6N5L1E2 (peak 23) decreased over time in the effluent (Supporting Information Fig. S-3E). The increase over time in effluent IgG glycans corresponds with the trend (Fig. 3A) in the total cohort and might reflect a higher level of inflammation.

The complete longitudinal dataset was assessed by logistic or linear regression in GEE analysis to reveal associations of the serum and effluent glycans with ten selected PD-related parameters with higher statistical power (N = 287 and 265 glycan mass spectra for serum and effluent, respectively). To this end, 12 derived glycan traits were tested against peritonitis occurrence, ultrafiltration during PET, D/P creatinine after 4 hours (PET), the concentrations of TGFβ-1, IL-6, IL-8, MCP-1, VEGF, CA-125 and HA. In addition, two presumably IgG-related single *N*-glycan compositions were tested for their association with peritonitis based on the trends found for the comparison of baseline vs. 24-months follow-up (Fig. 3C and D).

Variables known to have an impact on glycosylation, in particular age and sex^35^ as well as diabetes, were included in our statistical models, since they showed a tendency to correlate with glycan features in our baseline assessment of the data (Fig. 2, Supporting Information Fig. S-2A). We observed an area increase in H3N5F1 (peak 4) and a decrease in H5N4F1 (peak 5) with age in both serum and effluent, especially in women (Supporting Information Figure S-4). It is known that plasma protein galactosylation decreases with age in both healthy individuals and patients with different pathological conditions, which is mainly related to the galactosylation of IgG-derived glycans, including the two former-mentioned^41,43^. Possible confounders for PD-related parameters were also taken into account: treatment modality (CAPD or APD), time on PD and treatment group.

After adjustment for confounders, peritonitis occurrence, D/P creatinine after 4 hours (PET), TGFβ-1, IL-6, IL-8, MCP-1, VEGF, CA-125 and HA were associated with various glycan traits (Supporting Information Table S-3A and S-3B), as presented in the following paragraphs and summarised in Table 3. The association of ultrafiltration during PET with derived glycan traits was not significant after adjusting for confounders (Supporting Information Table S-3B).

**Table 3:** Overview of the main associations of clinical parameters with *N*-glycans.

Glycan trait		Peritonitis	DPCrea4	IL-6	IL-8	MCP-1	HA	TGFβ-1		VEGF	CA-125
Sample_Trait	Description	OR (95% CI)	Regression coefficient B (95% CI)								
Se_A2	Relative abundance of diantennary species (A2)	^a)^	^a)^	*−0.065* ^1^ *(−0.108; −0.022)*	*−0.061* ^1^ *(−0.114; −0.008)*	^a)^	^a)^	*−0.037°* *(−0.071; −0.002)*	*−0.089°* *(−0.141; −0.037)*		^a)^
Ef_A2		^a)^	*−0.008°* *(−0.016; −0.001)*	^a)^	*−0.101* ^3^ *(−0.190; −0.013)*	^a)^	^a)^	*−0.055°* *(−0.091; −0.020)*	^a)^		^a)^
Se_A3	Relative abundance of triantennary species (A3)	^a)^	^a)^	**0.077**° **(0.028; 0.125)**	**0.073**° **(0.011; 0.136)**	^a)^	^a)^	**0.039**° **(0.003; 0.076)**	**0.093**° **(0.038; 0.148)**		^a)^
Ef_A3		^a)^	**0.013** ^2^ **(0.004; 0.022)**	**0.121** ^2^ **(0.032; 0.210)**	**0.140** ^3^ **(0.029; 0.252)**	^a)^	^a)^	**0.083**° **(0.037; 0.129)**	**0.123** ^2^ **(0.028; 0.217)**		^a)^
Ef_A2F	Relative fucosylation of A2	**1.047** ^3^ **(1.000; 1.097)**	^a)^	^a)^	^a)^	^a)^	^a)^	^a)^	^a)^		*−0.022°* *(−0.042; −0.002)*
Se_A3F	Relative fucosylation of A3	^a)^	^a)^	^a)^	**0.016** ^1^ **(0.000; 0.032)**	^a)^	^a)^	^a)^	^a)^		^a)^
Ef_A3F		^a)^	^a)^	**0.026** ^0^ **(0.012; 0.040)**	^a)^	^a)^	^a)^	^a)^	^a)^		^a)^
Se_A2B	Relative bisection of A2	^a)^	^a)^	^a)^	**0.068** ^3^ **(0.001; 0.135)**	^a)^	^a)^	^a)^	^a)^		^a)^
Ef_A2B		^a)^	^a)^	^a)^	**0.076** ^3^ **(0.002; 0.151)**	^a)^	^a)^	^a)^	^a)^		
Se_A2G	Relative galactosylation of A2	^a)^	^a)^	^a)^	^a)^	^a)^	^a)^	*−0.028* ^2^ *(−0.053; −0.004)*	^a)^		^a)^
Ef_A2G		*0.871* ^3^ *(0.784; 0.967)*	^a)^	^a)^	^a)^	^a)^	^a)^	^a)^	^a)^		**0.045** ^2^ **(0.016; 0.074)**
Ef_A2L	Relative α2,3-*N-*sialylation per antenna within A2	^a)^	*−0.034* ^1^ *(−0.064; −0.004)*	*−0.403* ^3^ *(−0.723; −0.082)*	^a)^	^a)^	^a)^	^a)^	^a)^		^a)^
Ef_A3L	Relative α2,3-*N-*sialylation per antenna within A3	^a)^	^a)^	^a)^	**0.080** ^3^ **(0.015; 0.144)**	^a)^	^a)^	^a)^	^a)^		^a)^
Ef_A2E	Relative α2,6-*N-*sialylation per antenna within A2	*0.934* ^3^ *(0.876; 0.996)*	^a)^	^a)^	^a)^	^a)^	^a)^	^a)^	^a)^		**0.033** ^0^ **(0.009; 0.056)**
Se_A3E	Relative α2,6-*N-*sialylation per antenna within A3	^a)^	^a)^	**0.088** ^0^ **(0.020; 0.157)**	^a)^	^a)^	^a)^	^a)^	^a)^		^a)^
Ef_A3E		**1.205** ^0^ **(1.004; 1.446)**	**0.005** ^1^ **(0.001; 0.010)**	**0.113** ^0^ **(0.057; 0.169)**	**0.062** ^1^ **(0.008; 0.117)**	**0.041** ^0^ **(0.012; 0.070)**	**0.049** ^0^ **(0.023; 0.076)**	**0.052** ^3^ **(0.026; 0.079)**	**0.082** ^0^ **(0.044; 0.121)**		^a)^
Ef_IgG	Relative abundance of primarily IgG-derived glycans	**1.116** ^3^ **(1.017; 1.225)**	^a)^	^a)^	^a)^	^a)^	^a)^	^a)^	^a)^		*−0.045* ^0^ *(−0.072; −0.018)*
Ef_H3N4F1	Relative abundance of H3N4F1	**1.297** ^3^ **(1.098; 1.532)**	---	---	---	---	---	---	---		---
Ef_H3N5F1	Relative abundance of H3N5F1	**2.156** ^1^ **(1.192; 3.898)**	---	---	---	---	---	---	---		---

Displayed are odds ratios (OR) derived from logistic regression and regression coefficients B from linear regression along with their 95% confidence intervals, both using GEE for a robust estimation of the standard errors. Only associations with p < 0.05 and a 10%-cutoff for the change in estimate by confounders are shown, while associations not matching these criteria are listed in Supporting Information Table S-3 (here marked with ^a)^). Thus, high-mannose trait (M) in either serum or effluent, in addition to A2F, A2L, A3L, A2E and IgG in serum are not listed. Associations for direct traits with the compositions H3N4F1 and H3N5F1 were only tested for effluent, but not serum. Se, glycans from serum; Ef, glycans from PD effluent; H, hexose; N, *N*-acetylhexosamine; F, fucose.

The following models were used: ^0^, crude analysis; ^1^, model 1 with adjustment for age, sex, diabetes; ^2^, model 2 with adjustment for CAPD/APD, time on PD and Dianeal/Physioneal group; ^3^, model 1 + 2. Relative signal intensities of the derived glycan traits were calculated as %-values, and levels of IL-6, IL-8, MCP-1, HA, TGFβ-1, VEGF and CA-125 were ln-transformed prior to regression analysis. Negative and positive associations are formatted in *italic* and **bold**, respectively; not tested associations are depicted as [---]. For calculations of derived trait abundances, see Supporting Information Table S-1 and S-2.

#### Peritonitis occurrence

Peritonitis occurrence was positively associated with the relative abundance of IgG-derived glycans as well as the relative fucosylation of diantennary glycans (A2F) and α2,6-sialylation of triantennary glycans (A3E) in PDE only (excerpt in Table 3 and extensive information in Supporting Information Table S-3A). A negative association was found for the relative galactosylation (A2G) and α2,6-sialylation (A2E) of diantennary glycans. These associations are in line with the trends we observed towards study end, i.e. increasing A2F, IgG-glycans, A3E, and decreasing A2G and A2E (Fig. 3A). This strengthens the hypothesis that these *N-*glycan traits may reflect local peritoneal inflammation in PD patients, in particular, when assessed in PDE, since derived glycan traits in serum were not associated with peritonitis (not shown). When analysing a subselection of direct traits based on our findings for baseline and longitudinal glycan data, we found no association of selected triantennary α2,6-sialylated traits (peaks 16, 21, 23–24, 26) with peritonitis (data not shown). However, the two agalactosylated IgG-related structures (peaks 2 and 4), known for reflecting a pro-inflammatory state in other disease conditions^41,42^, were positively associated with peritonitis. These *N*-glycans are also the main contributors to the inverse association of galactosylation of diantennary glycans (A2G) with peritonitis (Table 3; Supporting Information Table S-3A).

PD effluent contains immunoglobulins, such as IgG and IgM^44^, which might reflect local inflammation during peritonitis^45^, as indicated by the positive association of peritonitis with effluent but not serum glycans. In addition, protein losses are known to increase during peritonitis, mainly due to enhanced peritoneal membrane permeability caused by inflammation^22,44^. In a proteomic study of PDE collected from twelve patients before and after peritonitis, several proteins were differentially expressed. For example, fibrinogen, ceruloplasmin, zinc-α-2-glycoprotein and α-1-antitrypsin were downregulated during peritonitis, whereas haptoglobin and antithrombin-III were upregulated^46^. Haptoglobin and alpha1-acid-glycoprotein are acute-phase proteins that contribute to the pool of triantennary plasma *N-*glycans with α2,6-sialylation^36^, and changes in their levels and glycosylation might also be the major cause for the positive association of the respective glycan trait (A3E) with peritonitis. Notably, in our global approach we were not able to trace back the origin of the relevant glycan traits to their protein carriers. However, as for the diantennary, fucosylated, non-sialylated glycans in the serum and effluent of PD patients, we assume that these are largely IgG-derived as we deduce from previous studies available on human serum and plasma glycomes^36^.

#### Inflammation markers and peritoneal function parameters

All tested inflammatory markers were positively associated with the degree of triantennary α2,6-sialylation of effluent proteins (EfA3E, Table 3). At the same time, the relative abundance of triantennary glycans (A3) and the degree of fucosylation of triantennary glycans (A3F) in serum and/or effluent was positively correlated with the IL-6 and IL-8 concentration in the effluent. Core and antenna fucosylation of acute-phase proteins has been shown to be increased in various disease conditions, such as chronic pancreatitis^47^, rheumatoid arthritis^48^ and inflammation related to cancer^49^. Furthermore, literature demonstrates that α2,6-sialyltransferase expression can be induced by different pro-inflammatory cytokines in human endothelial cells^50^. This enzyme was furthermore secreted into the medium^50^, indicating that α2,6-sialylation of circulating glycoproteins may occur *in vivo* upon inflammation.

Previously, various proteins were found to be differentially expressed in PD patients with low and high values of peritoneal transport, including glycoproteins such as haptoglobin, alpha-1 antitrypsin, and immunoglobulins^26,51^. Moreover, protein losses in PDE were found to be higher in high-transport patients as defined by higher D/P creatinine after 4 hours (PET) values^51^. Therefore, we analysed a possible association of serum and effluent protein glycosylation with transport characteristics in PD. Ultrafiltration was not associated with glycan traits after adjustment for confounders (Supporting Information Table S-3B), while D/P creatinine after 4 hours (PET) was positively associated with triantennary glycans and the α2,6-sialylation of those, and inversely associated with diantennary glycans and α2,3-sialylation of those in the effluent, but not serum (Table 3). The same glycan traits were associated with TGFβ-1 and VEGF concentrations (Table 3), which have been linked to pathological changes of the peritoneum upon PD^18^. Importantly, TGFβ-1-induced EMT in the context of tumour progression was found to be promoted by an increased expression of α2,6-sialyltranferase 1^52^. A similar mechanism might have led to the predominance of α2,6-sialylation of effluent *N-*glycans regarding its associations with detrimental PD markers/parameters in our data.

Furthermore, galactosylation of diantennary glycans was inversely correlated with effluent TGFβ-1 concentrations, but positively correlated with CA-125 that is considered as a marker of mesothelial cell mass, which is decreasing in PD patients developing complications, such as encapsulating peritoneal sclerosis^53^. Galactosylation of serum diantennary *N-*glycans was shown to be decreased in liver cirrhosis^29^, a pathological condition preceded by fibrotic tissue transformation that might show similarities to the pathological changes upon long-time PD. Both, a higher α2,6-sialylation of diantennary glycans (A2E) and a higher galactosylation of diantennary glycans in effluent with, at the same time, lower abundance of IgG-glycans seems to reflect a better clinical state, as indicated by their positive associations with CA-125 and inverse associations with peritonitis (Table 3).

#### Summary

As demonstrated by using a recently developed MALDI-TOF-MS method with linkage-specific sialic acid derivatisation, α2,6-sialylation of triantennary *N-*glycans in PD effluent seems to reflect adverse events upon long-term PD. Furthermore, a relative increase in IgG-related glycans and a lower galactosylation of diantennary glycans appeared to be related to peritonitis and the loss of mesothelial cell mass. Thus, glycosylation of effluent proteins in PD patients might bear some potential as future biomarkers of peritoneal functionality, hopefully aiding the detection of early-stage functionality changes – already before cytokine secretion and a destructive inflammatory cascade would take place. However, future research should include studies of the respective protein carriers, such as IgG, acute-phase proteins originating from the liver, or specific glycoproteins locally produced in the peritoneum, e.g. CA-125.

## Methods

### Study population and data collection

The current study is embedded in an open label multi-centre prospective randomised clinical trial, which enrolled 94 PD patients (aged over 18 years) from twelve different hospitals in the Netherlands during a period of 24 months (EudraCT 2006-001570-25). The study was approved by the medical ethics committee of the VU University Medical Center (‘medisch ethische toetsingscommissie van VUmc’), and was confirmed at all participating centres (see ref.^31^ for more details). The methods were carried out in accordance with the relevant guidelines and regulations (METC protocol number 2005.183). All study participants gave written informed consent.

Patients included in the randomised part of the study were treated with standard lactate-buffered PD fluid (Dianeal®, Baxter Healthcare, USA), and either continued on Dianeal (group 1, n = 38), or switched to a bicarbonate/lactate-buffered PD fluid (Physioneal®, Baxter Healthcare, USA; group 2, n = 40). A third group of patients (group 3, n = 16) was not included in the randomised part of the study and was already treated with Physioneal before the study period. They had never used Dianeal before. Prior to study start all patients had to undergo PD treatment for at least 6 weeks. Episodes of peritonitis or exit site infections that occurred within 6 weeks before entering the study, or use of non-glucose based dialysis solutions, except icodextrin, were considered exclusion criteria. Block randomisation per centre was performed centrally at the VU University Medical Center (4 patients per block and 2 patients assigned to each treatment per block).

### Sample preparation and measurement

Serum and 24-hours (overnight) dialysate samples were collected at 0, 6, 12, 18 and 24 months and stored at -80 °C until laboratory analysis.

#### Peritoneal Equilibrium Test (PET)

A 4-hour PET using 3.86% Dianeal or Physioneal was performed at 0, 12 and 24 weeks in order to assess peritoneal membrane function^54^. If a patient did not tolerate the 3.86% solution because of hypotension, 2.27% was used. Serum and effluent creatinine levels were determined using a cobas® 8000 immunoanalyser (Roche Diagnostics, Rotkreuz, Switzerland).

#### Peritonitis events

Peritonitis events were recorded and defined as a dialysate having a cell count higher than 100/μL, of which more than 50% were polymorphonuclear leukocytes. Effluents were cultured to define the microorganisms involved, and for the diagnosis also symptoms such as abdominal pain, fever and/or cloudy dialysate were taken into account^55^. Peritonitis relapses (peritonitis with the same organism or sterile episode occurring within 4 weeks of an earlier episode) were counted as 1 episode.

#### Analysis of effluent cytokines and serum lipids

Assays for cytokine detection were performed in cell-free supernatants of effluents collected at 0, 6, 12, 18 and 24 months. A multiparameter Bio-Plex 200 chemiluminometric bead assay kit (Biorad, Hercules, California, Texas, USA) was used to measure VEGF, IL-6, IL-8, and MCP-1 levels. HA was determined in an ELISA-based assay according to Fosang *et al*.^56^ using immobilised HA and competition for the binding of biotinylated HA-binding protein by HA-containing samples. TGFβ-1 was measured using a quantitative ELISA (Promega Corporation, Madison, USA, detection limit 32 pg/mL). A two-side sandwich immunoassay using direct chemiluminometric technology (Centaur OV-assay Bayer Diagnostics, Tarrytown, NY, USA) was used to determine CA-125 levels.

HsCRP was measured using a sensitive, enzyme-linked assay able to detect values below the detection limit of standard assays (DakoCytomation, Glostrup, Denmark). HbA1c was measured using the HA-8160 (Adams A1c) analyser which uses an HPLC cation exchange method (Menarini Diagnostics Benelux, Valkenswaard, The Netherlands). Serum lipids were measured by a cobas® 8000 immunoanalyser (Roche Diagnostics, Rotkreuz, Switzerland).

#### Sample treatment and glycomic analysis

*PVDF membrane N-glycan release*: *N-*glycans were released from the protein fraction on a protein-binding hydrophobic Immobilon-P Membrane (PVDF) plate (Merck Millipore, Darmstadt, Germany)^57^. Throughout the glycomic sample preparation and analysis, ultrapure water (MQ) was used (≥18.2 MΩ at 25 °C, Merck Millipore). The membrane was preconditioned by washing 2× with 200 µL 70% ethanol followed by two washes with 200 µL 100 mM NaHCO_3_. Biological samples were applied by mixing 72.5 µL 8 M GuHCl with 2.5 µL 200 mM dithiothreitol with 25 µL sample (2.5 times diluted serum and undiluted PDE) on the membrane and washing 4× with 200 µL buffer. Subsequently, 1 mU recombinant peptide-*N-*glycosidase F (PNGase F; Roche Diagnostics, Mannheim, Germany) was applied to the sample in 50 µL 100 mM NaHCO_3_ buffer, followed by overnight-incubation at 37 °C in a humidity chamber. The released *N*-glycans were recovered by centrifugation at 2000 rpm.

*Derivatisation for MALDI-TOF-MS*: The released glycans were derivatised for a selective ethyl-esterification of α2,6-linked *N-*acetylneuraminic acids and lactonisation of α2,3-linked *N-*acetylneuraminic acids^32^. The derivatisation mixture was prepared by mixing 1-ethyl-3-(3-dimethylaminopropyl)carbodiimide (Fluorochem, Hadfield, UK) with 1-hydroxybenzotriazole (Sigma-Aldrich, Steinheim, Germany), to a final concentration of 0.25 M in ethanol (Merck, Darmstadt, Germany). The reaction was performed in 20 μL of the derivatisation mixture with 1 µL released glycans from serum and 2 µL released glycans from PDE. The plate was sealed to prevent evaporation and incubated 1 h at 37 °C. To allow protein precipitation 20 µL of acetonitrile (Biosolve, Valkenswaard, the Netherlands) was added, further incubated for 15 min at −20 °C before proceeding with glycan enrichment and analysis by matrix-assisted laser desorption/ionisation time-of-flight mass spectrometry (MALDI-TOF-MS).

*Hydrophilic-interaction liquid-chromatographic purification for MALDI-TOF-MS*: Glycan enrichment was performed by cotton hydrophilic-interaction liquid-chromatography as previously described^58^. The samples were allowed to return to room temperature before proceeding. The tips with cotton as stationary phase (3 mm cotton thread, ca. 190 µg; Pipoos, Utrecht, the Netherlands) were washed and equilibrated with 3 × 20 μL of MQ and 3 × 20 μL of 85% acetonitrile; sample loading was achieved by pipetting 20× into the reaction mixture, the washing step consisted of pipetting 3 × 20 μL 85% acetonitrile 1% trifluoroacetic acid (Merck), 3 × 20 μL 85% acetonitrile. Samples were finally eluted in 10 μL MQ.

*MALDI-TOF-MS measurement*: For MALDI-TOF-MS analysis, 1 μL of purified serum- and 2 µL of purified PDE glycan solution was spotted on a MTP AnchorChip 800/384 TF MALDI target (Bruker Daltonics, Bremen, Germany), and 1 μL of matrix (5 mg/mL 2,5-dihydroxybenzoic acid (Bruker Daltonics), 1 mM NaOH in 50% acetonitrile), mixed on plate and left to dry. To achieve uniform crystals, the spot was tapped with 0.2 μL ethanol, causing rapid recrystallisation. All analyses were performed on an ultrafleXtreme MALDI-TOF/TOF-MS equipped with a Smartbeam II laser, controlled by proprietary software flexControl 3.4 Build 119 (Bruker Daltonics). The MALDI-TOF instrument was operated in reflectron positive ion mode, calibrated on the known masses of a peptide calibration standard (Bruker Daltonics). For sample measurements 20,000 laser shots were accumulated at a laser frequency of 2,000 Hz, using a complete-sample random walk with 200 shots per raster spot.

### Data processing

The mass spectra were extracted and processed for quality control and relative quantitation of analytes. The raw spectra were exported from flexAnalysis 3.4 (Build 76; Bruker Daltonics) as text files (x,y), and further processed by MassyTools (version 0.1.8.0)^59^. First, internal calibration was performed based on a predefined list of 5 analytes (peaks 2, 3, 5, 9, 18 in Supporting Information Table S-1). Mass spectra presenting a signal-to-noise ratio (S/N) of 9 or above for the calibration analytes were included for further analysis (S/N based on the MinMax algorithm). Furthermore, 10 spectra were excluded because their “fraction of analyte area above S/N 9” was below 3 standard deviations (SD) of the mean.

Structural assignment was based on the putative compositional features (hexose = H, *N-*acetylhexosamine = N, fucose = F, *N-*acetylneuraminic acid = E or L for α2,6- and α2,3-linked variants, respectively). After the exclusion of compositions with interfering peaks (n = 15), background-subtracted peak areas were normalised to the sum of the areas of all the 26 peaks from the final list of 26 glycan compositions. Derived traits were calculated based on the compositional features based on established knowledge of the human plasma *N-*glycome and its biosynthetic pathways (Supporting Information Table S-1 and S-2)^32–34^. For example, bisection of diantennary glycans was assumed rather than an agalactosylated third antenna for H5N5E1F1. Finally, relative intensities were multiplied by the factor of 100 prior to statistical analysis.

### Statistical analysis

Effluent marker levels, used as dependent variables, were ln-transformed prior to statistical analysis due to their non-normal distribution. When generating association heatmaps, derived glycan trait values were scaled (mean subtraction and division by SD) to obtain interpretable estimates, i.e. t-statistics and Wald statistics in case of linear and logistic regression, respectively.

The generation of heatmaps for baseline data was carried out by using the ‘rquery.cormat’ function and ‘ggplot2’ and ‘WGCNA’ libraries^60^. To this end, R version 3.2.2 in an RStudio environment was employed (RStudio Team, Boston, MA).

Paired Wilcoxon signed-rank tests (two-tailed) were performed in IBM SPSS Statistics 23 to compare serum vs. effluent glycosylation levels at baseline as well as to compare baseline vs. 24-months levels of glycan traits that showed changing trends towards the end of the study.

Analysis of the longitudinal data was further performed by using the Generalized Estimated Equations (GEE) method with an exchangeable working correlation matrix in IBM SPSS Statistics 23. By using ‘individual’ as the grouping variable, multiple measurements within an individual were taken into account, providing more robust data. A binary logistic GEE model was used for the evaluation of the association of glycan traits with peritonitis occurrence, while a linear model was applied to estimate the association of glycan traits with effluent markers and peritoneal transport parameters, i.e. the (log-transformed) concentration of TGFβ-1, IL-6, IL-8, MCP-1, VEGF, CA-125 and HA, as well as ultrafiltration during PET and D/P creatinine after 4 hours (PET). Next to performing crude GEE-analysis, we adjusted for confounders in three models: model 1: age, sex and diabetes; model 2: treatment modality (CAPD or APD), time on PD and treatment group; model 3: a combination of all confounders of model 1 and model 2. Age and time on PD were added as continuous variables, whereas sex, diabetes, treatment modality, and treatment group were added as categorical variables. Regression coefficients and odds ratios are given with 95% confidence intervals.

### Data availability

The extracted glycomics data including all time points available per patient is compiled in Supporting Information Table S-4. The MS glycomics raw data generated and analysed during the current study as well as the clinical data from individuals are available from the corresponding author on reasonable request.

## Electronic supplementary material

Supplementary Tables S1-3 and Figures S1-4

Supplementary Table S4

## References

[1] Jain AK, Blake P, Cordy P, Garg AX (2012). Global trends in rates of peritoneal dialysis. J Am Soc Nephrol.

[2] Schilte MN, Celie JW, Wee PM, Beelen RH, van den Born J (2009). Factors contributing to peritoneal tissue remodeling in peritoneal dialysis. Peritoneal dialysis international: journal of the International Society for Peritoneal Dialysis.

[3] Lai KN, Tang SC, Leung JC (2007). Mediators of inflammation and fibrosis. Peritoneal dialysis international: journal of the International Society for Peritoneal Dialysis.

[4] Williams JD (2002). Morphologic changes in the peritoneal membrane of patients with renal disease. J Am Soc Nephrol.

[5] Kalluri R, Weinberg RA (2009). The basics of epithelial-mesenchymal transition. J Clin Invest.

[6] Thiery JP, Sleeman JP (2006). Complex networks orchestrate epithelial-mesenchymal transitions. Nat Rev Mol Cell Biol.

[7] Smit W, Parikova A, Krediet RT (2005). Ultrafiltration failure in peritoneal dialysis. Causes and clinical consequences. Minerva Urol Nefrol.

[8] Lamouille S, Xu J, Derynck R (2014). Molecular mechanisms of epithelial-mesenchymal transition. Nat Rev Mol Cell Biol.

[9] Xu J, Lamouille S, Derynck R (2009). TGF-beta-induced epithelial to mesenchymal transition. Cell Res.

[10] Leask A, Abraham DJ (2004). TGF-beta signaling and the fibrotic response. FASEB J.

[11] Tan Z (2014). Altered N-Glycan expression profile in epithelial-to-mesenchymal transition of NMuMG cells revealed by an integrated strategy using mass spectrometry and glycogene and lectin microarray analysis. Journal of proteome research.

[12] Maupin KA (2010). Glycogene expression alterations associated with pancreatic cancer epithelial-mesenchymal transition in complementary model systems. PLoS One.

[13] Krediet RT (2001). Dialysate cancer antigen 125 concentration as marker of peritoneal membrane status in patients treated with chronic peritoneal dialysis. Peritoneal dialysis international: journal of the International Society for Peritoneal Dialysis.

[14] Yung S, Chan TM (2011). Pathophysiology of the peritoneal membrane during peritoneal dialysis: the role of hyaluronan. J Biomed Biotechnol.

[15] Margetts PJ (2001). Gene transfer of transforming growth factor-beta1 to the rat peritoneum: effects on membrane function. J Am Soc Nephrol.

[16] Liu J (2013). High-glucose-based peritoneal dialysis solution induces the upregulation of VEGF expression in human peritoneal mesothelial cells: The role of pleiotrophin. Int J Mol Med.

[17] Chow AW (2010). Polarized secretion of interleukin (IL)-6 and IL-8 by human airway epithelia 16HBE14o- cells in response to cationic polypeptide challenge. PLoS One.

[18] Lopes Barreto D, Krediet RT (2013). Current status and practical use of effluent biomarkers in peritoneal dialysis patients. Am J Kidney Dis.

[19] Yang X (2014). Intraperitoneal interleukin-6 levels predict peritoneal solute transport rate: a prospective cohort study. Am J Nephrol.

[20] Krediet RT (2013). Peritoneal dialysis: from bench to bedside. Clin Kidney J.

[21] Perl J, Nessim SJ, Bargman JM (2011). The biocompatibility of neutral pH, low-GDP peritoneal dialysis solutions: benefit at bench, bedside, or both?. Kidney Int.

[22] Dulaney JT, Hatch FE (1984). Peritoneal dialysis and loss of proteins: A review. Kidney International.

[23] Cuccurullo M (2011). Proteomic analysis of peritoneal fluid of patients treated by peritoneal dialysis: effect of glucose concentration. Nephrol Dial Transplant.

[24] Raaijmakers R (2008). Proteomic profiling and identification in peritoneal fluid of children treated by peritoneal dialysis. Nephrol Dial Transplant.

[25] Yang MH (2013). Proteomic profiling for peritoneal dialysate: differential protein expression in diabetes mellitus. Biomed Res Int.

[26] Wen Q (2013). Proteomic analysis in peritoneal dialysis patients with different peritoneal transport characteristics. Biochem Biophys Res Commun.

[27] Ohtsubo K, Marth JD (2006). Glycosylation in cellular mechanisms of health and disease. Cell.

[28] Novokmet M (2014). Changes in IgG and total plasma protein glycomes in acute systemic inflammation. Scientific reports.

[29] Callewaert N (2004). Noninvasive diagnosis of liver cirrhosis using DNA sequencer-based total serum protein glycomics. Nature medicine.

[30] Rombouts, Y. *et al*. Acute phase inflammation is characterized by rapid changes in plasma/peritoneal fluid N-glycosylation in mice. *Glycoconj J*, 10.1007/s10719-015-9648-9 (2016). 10.1007/s10719-015-9648-9 PMC4891370 26924641

[31] Farhat, K. *et al*. Effects of Conversion to a Bicarbonate/Lactate-Buffered, Neutral-Ph, Low-Gdp Pd Regimen in Prevalent Pd: A 2-Year Randomized Clinical Trial. *Perit Dial Int*, 10.3747/pdi.2015.00031 (2017). 10.3747/pdi.2015.00031 28348100

[32] Reiding KR, Blank D, Kuijper DM, Deelder AM, Wuhrer M (2014). High-throughput profiling of protein N-glycosylation by MALDI-TOF-MS employing linkage-specific sialic acid esterification. Analytical chemistry.

[33] Royle L (2008). HPLC-based analysis of serum N-glycans on a 96-well plate platform with dedicated database software. Analytical biochemistry.

[34] Stumpo KA, Reinhold VN (2010). The N-glycome of human plasma. Journal of proteome research.

[35] Reiding KR (2017). Human Plasma N-glycosylation as Analyzed by Matrix-Assisted Laser Desorption/Ionization-Fourier Transform Ion Cyclotron Resonance-MS Associates with Markers of Inflammation and Metabolic Health. Molecular & Cellular Proteomics.

[36] Clerc, F. *et al*. Human plasma protein N-glycosylation. *Glycoconj J*, 10.1007/s10719-015-9626-2 (2015). 10.1007/s10719-015-9626-2 PMC4891372 26555091

[37] Bladergroen MR (2015). Automation of High-Throughput Mass Spectrometry-Based Plasma N-Glycome Analysis with Linkage-Specific Sialic Acid Esterification. Journal of proteome research.

[38] Jansen BC (2016). Pregnancy-associated serum N-glycome changes studied by high-throughput MALDI-TOF-MS. Scientific reports.

[39] Spaia S (1993). Variability of peritoneal protein loss in diabetic and nondiabetic patients on continuous ambulatory peritoneal dialysis. Perit Dial Int.

[40] Wu HY (2013). Comparative proteomic analysis of peritoneal dialysate from chronic glomerulonephritis patients. Biomed Res Int.

[41] Dall’Olio F (2013). N-glycomic biomarkers of biological aging and longevity: a link with inflammaging. Ageing research reviews.

[42] Parekh RB (1985). Association of rheumatoid arthritis and primary osteoarthritis with changes in the glycosylation pattern of total serum IgG. Nature.

[43] Borelli V (2015). Plasma N-Glycome Signature of Down Syndrome. Journal of proteome research.

[44] Blumenkrantz MJ (1981). Protein losses during peritoneal dialysis. Kidney Int.

[45] Cameron JS (1995). Host defences in continuous ambulatory peritoneal dialysis and the genesis of peritonitis. Pediatr Nephrol.

[46] Tyan YC, Su SB, Ting SS, Wang HY, Liao PC (2013). A comparative proteomics analysis of peritoneal dialysate before and after the occurrence of peritonitis episode by mass spectrometry. Clin Chim Acta.

[47] Ueda, M. *et al*. Specific increase in serum core-fucosylated haptoglobin in patients with chronic pancreatitis. *Pancreatology*, 10.1016/j.pan.2016.01.004 (2016). 10.1016/j.pan.2016.01.004 26897254

[48] Olewicz-Gawlik A, Korczowska-Lacka I, Lacki JK, Klama K, Hrycaj P (2007). Fucosylation of serum alpha1-acid glycoprotein in rheumatoid arthritis patients treated with infliximab. Clin Rheumatol.

[49] Peracaula R, Sarrats A, Rudd PM (2010). Liver proteins as sensor of human malignancies and inflammation. Proteomics Clin Appl.

[50] Hanasaki K, Varki A, Stamenkovic I, Bevilacqua MP (1994). Cytokine-induced beta-galactoside alpha-2,6-sialyltransferase in human endothelial cells mediates alpha 2,6-sialylation of adhesion molecules and CD22 ligands. J Biol Chem.

[51] Sritippayawan S (2007). Proteomic analysis of peritoneal dialysate fluid in patients with different types of peritoneal membranes. Journal of proteome research.

[52] Lu J (2014). Beta-Galactosidealpha2,6-sialyltranferase 1 promotes transforming growth factor-beta-mediated epithelial-mesenchymal transition. J Biol Chem.

[53] Sampimon DE (2010). Early diagnostic markers for encapsulating peritoneal sclerosis: a case-control study. Perit Dial Int.

[54] Smit W (2003). Peritoneal function and assessment of reference values using a 3.86% glucose solution. Peritoneal dialysis international: journal of the International Society for Peritoneal Dialysis.

[55] Li PK (2010). Peritoneal dialysis-related infections recommendations: 2010 update. Perit Dial Int.

[56] Fosang AJ, Hey NJ, Carney SL, Hardingham TE (1990). An ELISA plate-based assay for hyaluronan using biotinylated proteoglycan G1 domain (HA-binding region). Matrix.

[57] Burnina I, Hoyt E, Lynaugh H, Li H, Gong B (2013). A cost-effective plate-based sample preparation for antibody N-glycan analysis. Journal of chromatography. A.

[58] Selman MH, Hemayatkar M, Deelder AM, Wuhrer M (2011). Cotton HILIC SPE microtips for microscale purification and enrichment of glycans and glycopeptides. Analytical chemistry.

[59] Jansen BC (2015). MassyTools: A High-Throughput Targeted Data Processing Tool for Relative Quantitation and Quality Control Developed for Glycomic and Glycoproteomic MALDI-MS. Journal of proteome research.

[60] Langfelder P, Horvath S (2008). WGCNA: an R package for weighted correlation network analysis. BMC Bioinformatics.

