# Supplementary material for: Effluent and serum protein N-glycosylation is associated with inflammation and peritoneal membrane transport characteristics in peritoneal dialysis patients
Source: Sci Rep. 2018 Jan 17;8:979. doi: 10.1038/s41598-018-19147-x (PMC5772620; doi:10.1038/s41598-018-19147-x)
Supplement: Supplementary file 1 — Supplementary Tables S1-3 and Figures S1-4 [file 41598_2018_19147_MOESM1_ESM.pdf]

Supporting Information for the manuscript (to be published in Scientific Reports):

Effluent and serum protein *N*-glycosylation is associated with inflammation and peritoneal membrane transport characteristics in peritoneal dialysis patients

Evelina Ferrantelli[1,#], Karima Farhat[2], Agnes L. Hipgrave Ederveen[3], Karli R. Reiding[3], Robert H.J. Beelen[1], Frans J. van Ittersum[2], Manfred Wuhrer[1,3,4], and Viktoria Dotz[3,4*]

[1] VU University Medical Center, Department of Molecular Cell Biology and Immunology, Amsterdam, The Netherlands

[2] VU University Medical Center, Department of Nephrology, Amsterdam, The Netherlands

[3] Center for Proteomics and Metabolomics, Leiden University Medical Center, Leiden, The Netherlands

[4] VU University Amsterdam, Division of BioAnalytical Chemistry, Amsterdam, The Netherlands

[#] Current address: Academic Medical Center, Tytgat Institute for Gastrointestinal and Liver Disease, Amsterdam, The Netherlands

* Corresponding author: v.dotz@lumc.nl or v-dotz@t-online.de, phone: +31 (0)71 526 87 02

Contents

Supporting Information Figure S-1. Comparison of the PNGase F *N*-glycan release from peritoneal effluent samples using the conventional in-solution approach (A and B) and protein immobilisation on a PVDF membrane (C)…………………………………………………………………………..Page S-2

Supporting Information Table S-1. Detected glycan molecular species, their putative structures and related derived traits..Page S-3

Supporting Information Table S-2. Calculation of the relative abundances of 12 glycan traits derived from 26 distinct glycan species detected in MALDI-TOF-MS ....Page S-6

Supporting Information Figure S-2. Heatmaps of the associations of derived glycan traits in serum with clinical parameters (A) and correlations between serum and effluent glycans at baseline ……………………………………………………………………………………… …...Page S-8

Supporting Information Figure S-3. Relative areas of selected *N*-glycan traits at baseline and over time ……………………………………………………………………………………………….Page S-9

Supporting Information Figure S-4. Age and sex-dependency of *N*-glycans in serum and peritoneal effluent ……………………………………………………………………………………..…Page S-10

Supporting Information Table S-3A. Associations of peritonitis with glycans according to logistic regression analysis with GEE………………………………………………………………………Page S-11

Supporting Information Table S-3B. Associations of inflammatory and transport parameters with glycans according to linear regression analysis with GEE……………………………………Page S-12

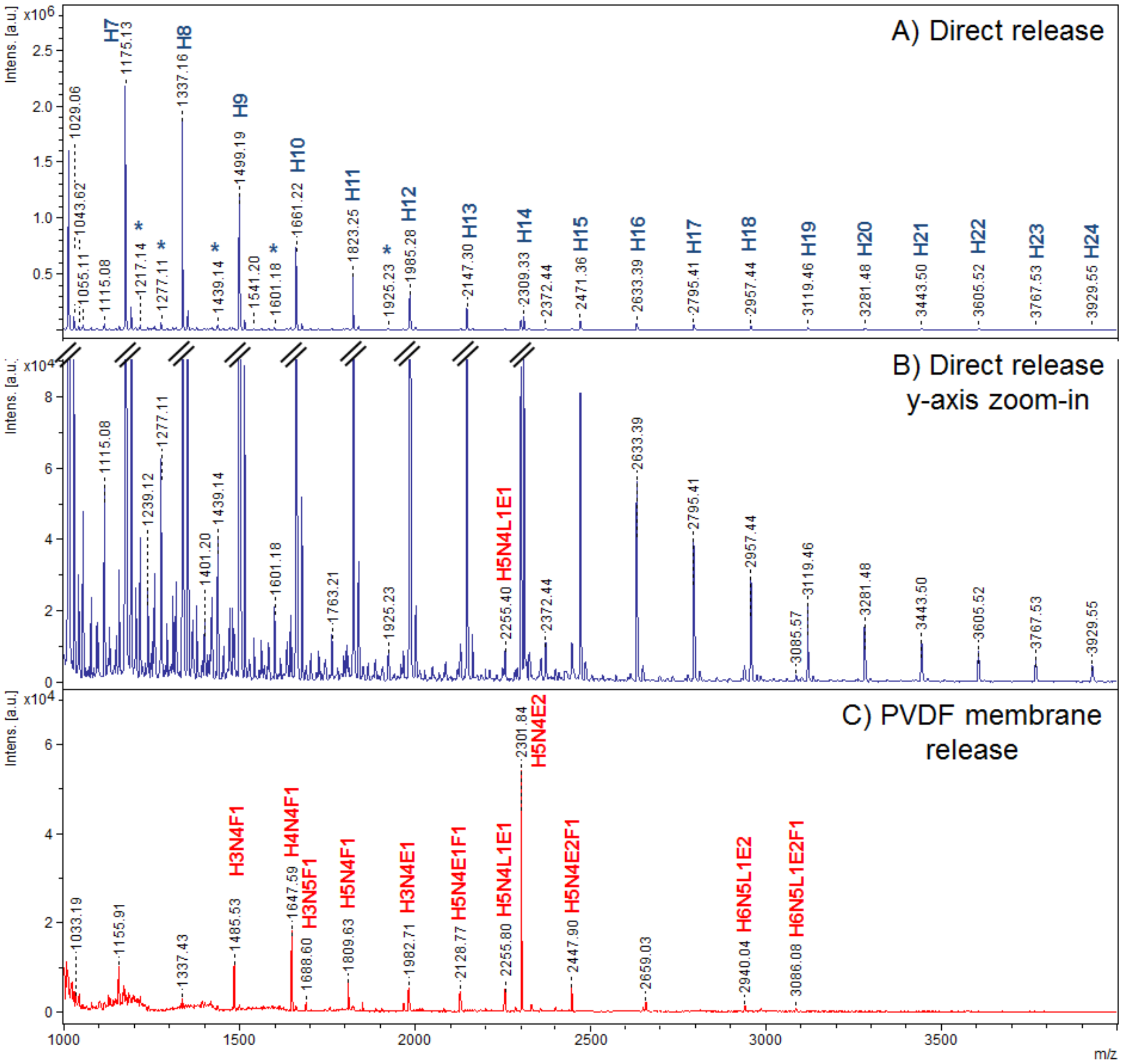

Supporting Information Figure S-1. Comparison of the PNGase F *N*-glycan release from peritoneal effluent samples using the conventional in-solution approach (A and B) and protein immobilisation on a PVDF membrane (C). Hexose polymers (blue font) originating from the peritoneal fluid applied to the patient dominate the mass spectrum (A and B), while *N*-glycan compositions (red font) are detected with very low intensity (B). When purifying the effluent after protein immobilization on a PVDF membrane, an *N*-glycan profile mostly free of interfering dextran peaks is acquired (C). H, hexose; N, *N*-acetylhexosamine; F, fucose; E, α2,6-*N*-acetylneuraminic acid; L, α2,3-*N*-acetylneuraminic acid; asterisks indicate cross-ring fragments of the dextran polymers.

Supporting Information Table S-1. Detected glycan molecular species, their putative structures and related derived traits

**Peak no.**	***m/z* [M+Na]$^+$**	**Composition** [a)]	**Proposed structure**	**Derived traits** [b)]											
				M	A2	A3	A2B	A2G	A2F	A3F	IgG	A2L	A3L	A2E	A3E
1	1419.48	H6N2	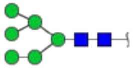	X											
2	1485.53	H3N4F1	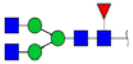		X				X		X				
3	1647.59	H4N4F1	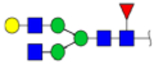		X			X	X		X				
4	1688.61	H3N5F1	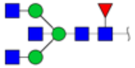		X		X		X		X				
5	1809.64	H5N4F1	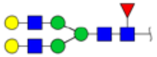		X			X	X		X				
6	1850.67	H4N5F1	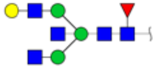		X		X	X	X		X				
7	1905.63	H9N2	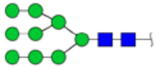	X											
8	1966.71	H4N4E1F1	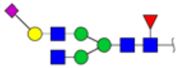		X			X	X					X	
9	1982.71	H5N4E1	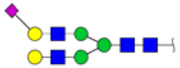		X			X						X	
10	2082.72	H5N4L1F1	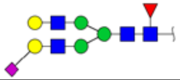		X			X	X			X			

11	2128.77	H5N4E1F1	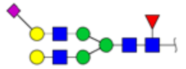		X			X	X					X	
12	2185.79	H5N5E1	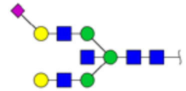		X		X	X						X	
13	2255.79	H5N4L1E1	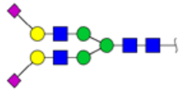		X			X				X		X	
14	2301.83	H5N4E2	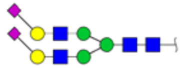		X			X						X	
15	2331.85	H5N5E1F1	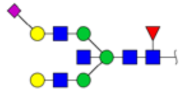		X		X	X	X					X	
16	2347.84	H6N5E1	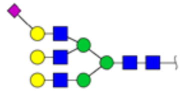			X									X
17	2401.85	H5N4L1E1F1	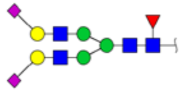		X			X	X			X		X	
18	2447.89	H5N4E2F1	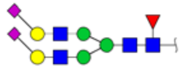		X			X	X					X	
19	2620.93	H6N5L1E1	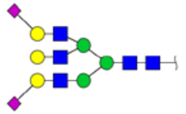			X							X		X
20	2650.97	H5N5E2F1	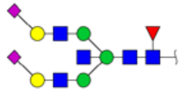		X		X	X	X					X	

21	2813.02	H6N5E2F1	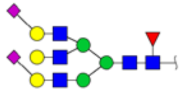			X				X					X
22	2894.01	H6N5L2E1	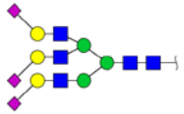			X							X		X
23	2940.05	H6N5L1E2	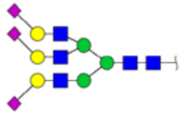			X							X		X
24	2986.09	H6N5E3	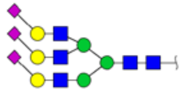			X									X
25	3040.07	H6N5L2E1F1	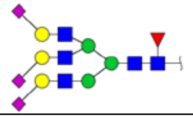			X				X			X		X
26	3086.11	H6N5L1E2F1	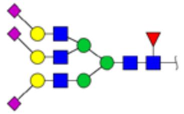			X				X			X		X

[a)] H, hexose; N, *N*-acetylhexosamine; F, fucose; E, α2,6-*N*-acetylneuraminic acid; L, α2,3-*N*-acetylneuraminic acid
[b)] M, high-mannose type; A2, diantennary; A3, triantennary; A2B, bisection of diantennary glycans; A2G, galactosylation of diantennary glycans; A2F, fucosylation of diantennary glycans; A3F, fucosylation of triantennary glycans; IgG, IgG-derived glycans; A2L, α2,3-*N*-acetylneuraminic acids per diantennary glycan; A3L, α2,3-*N*-acetylneuraminic acids per triantennary glycan; A2E, α2,6-*N*-acetylneuraminic acids per diantennary glycan; A3E, α2,6-*N*-acetylneuraminic acids per triantennary glycan; for calculation, see Table S-2.

Supporting Information Table S-2. Calculation of the relative abundances of 12 glycan traits derived from 26 distinct glycan species detected by MALDI-TOF-MS

	**Derived trait**	**Depiction**	**Calculation**
M	Relative abundance of high-mannose type species in spectrum	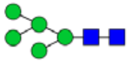	(1 * (H6N2 + H9N2)) / (1 * (H6N2 + H3N4F1 + H4N4F1 + H3N5F1 + H5N4F1 + H4N5F1 + H9N2 + H4N4E1F1 + H5N4E1 + H5N4L1F1 + H5N4E1F1 + H5N5E1 + H5N4L1E1 + H5N4E2 + H5N5E1F1 + H6N5E1 + H5N4L1E1F1 + H5N4E2F1 + H6N5L1E1 + H5N5E2F1 + H6N5E2F1 + H6N5L2E1 + H6N5L1E2 + H6N5E3 + H6N5L2E1F1 + H6N5L1E2F1))
A2	Relative abundance of diantennary species in spectrum	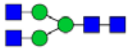	(1 * (H3N4F1 + H4N4F1 + H3N5F1 + H5N4F1 + H4N5F1 + H4N4E1F1 + H5N4E1 + H5N4L1F1 + H5N4E1F1 + H5N5E1 + H5N4L1E1 + H5N4E2 + H5N5E1F1 + H5N4L1E1F1 + H5N4E2F1 + H5N5E2F1)) / (1 * (H6N2 + H3N4F1 + H4N4F1 + H3N5F1 + H5N4F1 + H4N5F1 + H9N2 + H4N4E1F1 + H5N4E1 + H5N4L1F1 + H5N4E1F1 + H5N5E1 + H5N4L1E1 + H5N4E2 + H5N5E1F1 + H6N5E1 + H5N4L1E1F1 + H5N4E2F1 + H6N5L1E1 + H5N5E2F1 + H6N5E2F1 + H6N5L2E1 + H6N5L1E2 + H6N5E3 + H6N5L2E1F1 + H6N5L1E2F1))
A3	Relative abundance of triantennary species in spectrum	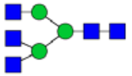	(1 * (H6N5E1 + H6N5L1E1 + H6N5E2F1 + H6N5L2E1 + H6N5L1E2 + H6N5E3 + H6N5L2E1F1 + H6N5L1E2F1)) / (1 * (H6N2 + H3N4F1 + H4N4F1 + H3N5F1 + H5N4F1 + H4N5F1 + H9N2 + H4N4E1F1 + H5N4E1 + H5N4L1F1 + H5N4E1F1 + H5N5E1 + H5N4L1E1 + H5N4E2 + H5N5E1F1 + H6N5E1 + H5N4L1E1F1 + H5N4E2F1 + H6N5L1E1 + H5N5E2F1 + H6N5E2F1 + H6N5L2E1 + H6N5L1E2 + H6N5E3 + H6N5L2E1F1 + H6N5L1E2F1))
A2B	Bisection of diantennary glycans	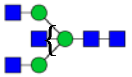	(1 * (H3N5F1 + H4N5F1 + H5N5E1 + H5N5E1F1 + H5N5E2F1)) / (1 * (H3N4F1 + H4N4F1 + H3N5F1 + H5N4F1 + H4N5F1 + H4N4E1F1 + H5N4E1 + H5N4L1F1 + H5N4E1F1 + H5N5E1 + H5N4L1E1 + H5N4E2 + H5N5E1F1 + H5N4L1E1F1 + H5N4E2F1 + H5N5E2F1))
A2G	Galactosylation per antenna within diantennary species	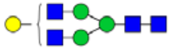	(1/2 * (H4N4F1 + H4N5F1 + H4N4E1F1) + 2/2 * (H5N4F1 + H5N4E1 + H5N4L1F1 + H5N4E1F1 + H5N5E1 + H5N4L1E1 + H5N4E2 + H5N5E1F1 + H5N4L1E1F1 + H5N4E2F1 + H5N5E2F1)) / (1 * (H3N4F1 + H4N4F1 + H3N5F1 + H5N4F1 + H4N5F1 + H4N4E1F1 + H5N4E1 + H5N4L1F1 + H5N4E1F1 + H5N5E1 + H5N4L1E1 + H5N4E2 + H5N5E1F1 + H5N4L1E1F1 + H5N4E2F1 + H5N5E2F1))
A2F	Relative abundance of fucosylated species within diantennary glycans	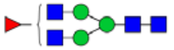	(1 * (H3N4F1 + H4N4F1 + H3N5F1 + H5N4F1 + H4N5F1 + H4N4E1F1 + H5N4L1F1 + H5N4E1F1 + H5N5E1F1 + H5N4L1E1F1 + H5N4E2F1 + H5N5E2F1)) / (1 * (H3N4F1 + H4N4F1 + H3N5F1 + H5N4F1 + H4N5F1 + H4N4E1F1 + H5N4E1 + H5N4L1F1 + H5N4E1F1 + H5N5E1 + H5N4L1E1 + H5N4E2 + H5N5E1F1 + H5N4L1E1F1 + H5N4E2F1 + H5N5E2F1))
A3F	Relative abundance of fucosylated species within triantennary glycans	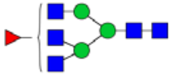	(1 * (H6N5E2F1 + H6N5L2E1F1 + H6N5L1E2F1)) / (1 * (H6N5E1 + H6N5L1E1 + H6N5E2F1 + H6N5L2E1 + H6N5L1E2 + H6N5E3 + H6N5L2E1F1 + H6N5L1E2F1))
IgG	Relative abundance of primarily IgG-derived glycans	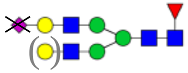	(H3N4F1 + H4N4F1 + H3N5F1 + H5N4F1 + H4N5F1)
A2L	α2,3-*N*-acetylneuraminic acid per antenna within diantennary glycans	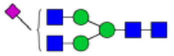	(1/2 * (H5N4L1F1 + H5N4L1E1 + H5N4L1E1F1) + 2/2 * (0)) / (1 * (H3N4F1 + H4N4F1 + H3N5F1 + H5N4F1 + H4N5F1 + H4N4E1F1 + H5N4E1 + H5N4L1F1 + H5N4E1F1 + H5N5E1 + H5N4L1E1 + H5N4E2 + H5N5E1F1 + H5N4L1E1F1 + H5N4E2F1 + H5N5E2F1))

A3L	α2,3-*N*-acetylneuraminic acid per antenna within triantennary glycans	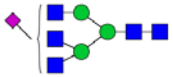	(1/3 * (H6N5L1E1 + H6N5L1E2 + H6N5L1E2F1) + 2/3 * (H6N5L2E1 + H6N5L2E1F1) + 3/3 * (0)) / (1 * (H6N5E1 + H6N5L1E1 + H6N5E2F1 + H6N5L2E1 + H6N5L1E2 + H6N5E3 + H6N5L2E1F1 + H6N5L1E2F1))
A2E	α2,6-*N*-acetylneuraminic acid per antenna within diantennary glycans	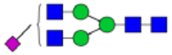	(1/2 * (H4N4E1F1 + H5N4E1 + H5N4E1F1 + H5N5E1 + H5N4L1E1 + H5N5E1F1 + H5N4L1E1F1) + 2/2 * (H5N4E2 + H5N4E2F1 + H5N5E2F1)) / (1 * (H3N4F1 + H4N4F1 + H3N5F1 + H5N4F1 + H4N5F1 + H4N4E1F1 + H5N4E1 + H5N4L1F1 + H5N4E1F1 + H5N5E1 + H5N4L1E1 + H5N4E2 + H5N5E1F1 + H5N4L1E1F1 + H5N4E2F1 + H5N5E2F1))
A3E	α2,6-*N*-acetylneuraminic acid per antenna within triantennary glycans	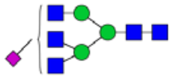	(1/3 * (H6N5E1 + H6N5L1E1 + H6N5L2E1 + H6N5L2E1F1) + 2/3 * (H6N5E2F1 + H6N5L1E2 + H6N5L1E2F1) + 3/3 * (H6N5E3)) / (1 * (H6N5E1 + H6N5L1E1 + H6N5E2F1 + H6N5L2E1 + H6N5L1E2 + H6N5E3 + H6N5L2E1F1 + H6N5L1E2F1))

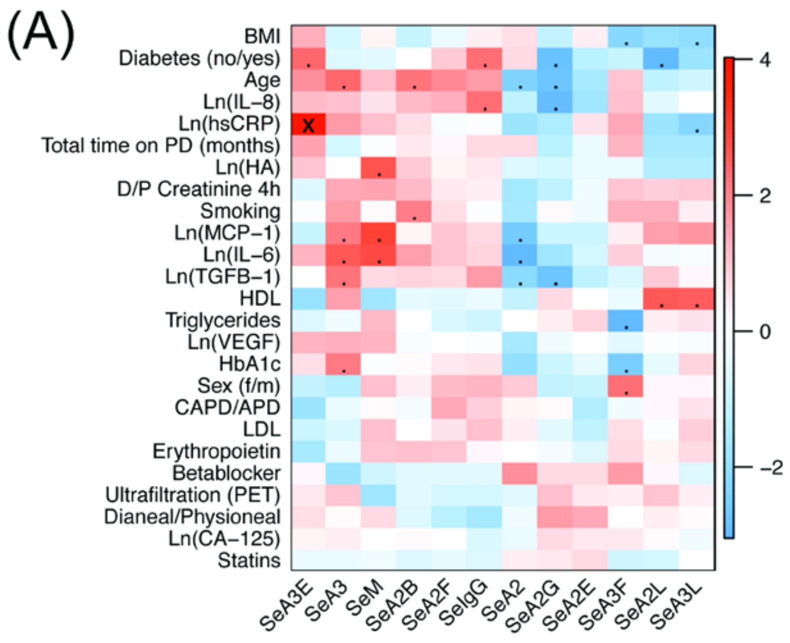

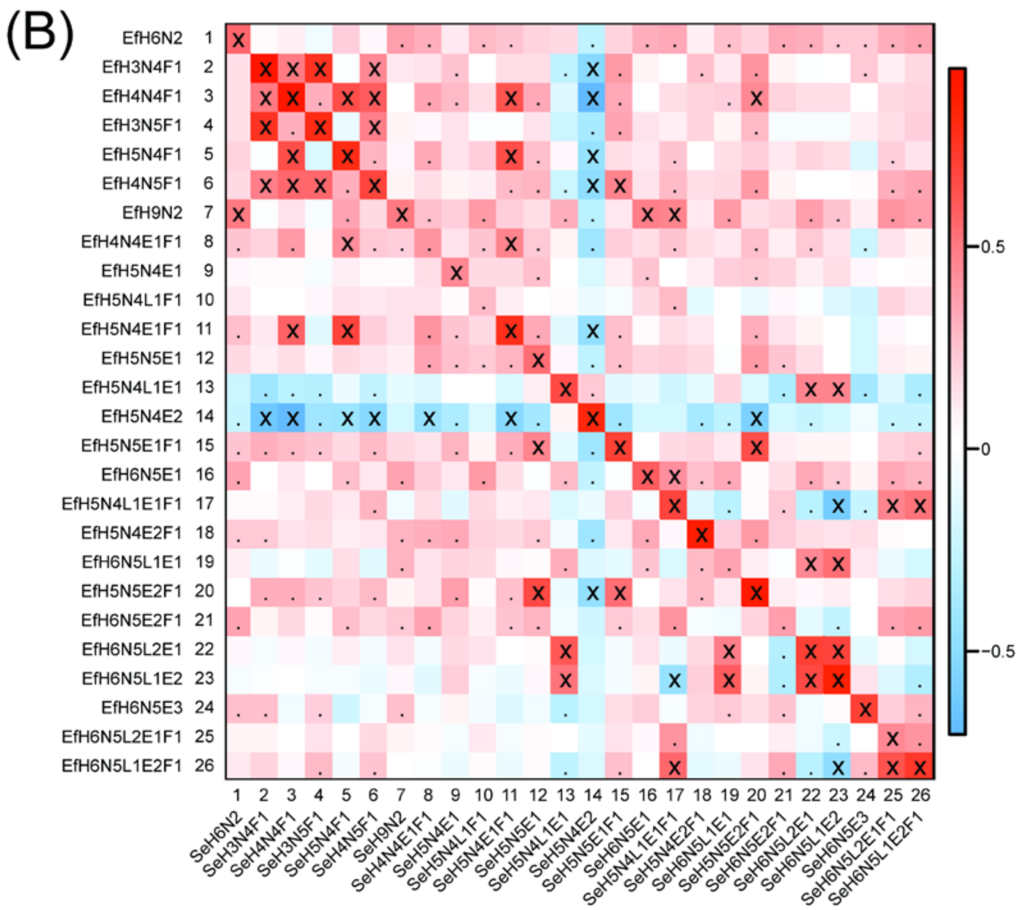

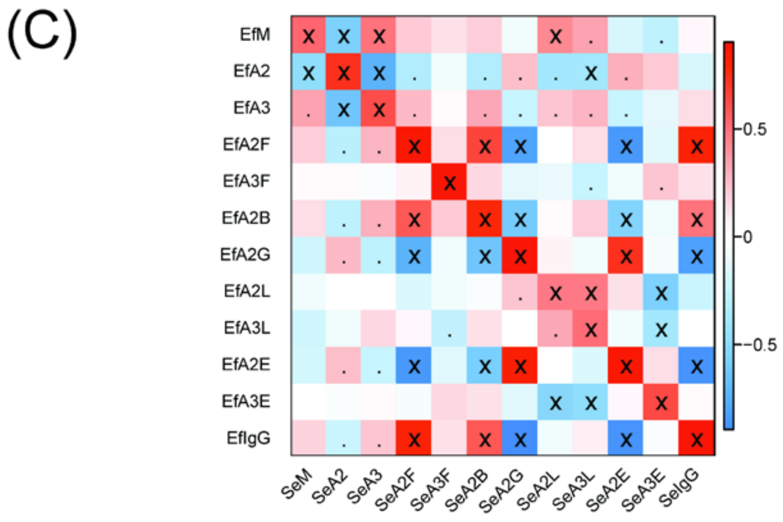

Supporting Information Figure S-2. Associations of derived glycan traits in serum with clinical parameters (A) and correlations between serum and effluent glycans at baseline. Depicted are t- or Wald statistics (β/SE) upon hierarchical clustering in (A) and Spearman correlation coefficients in (B) and (C). For details on glycan structures and derived traits, see Supporting Information Table S-1 and S-2. The ranking of the categorical variables in (A) is matching the listing order of their categories, e.g. for Sex, female = 0, male = 1; for the binary variables, i.e. Diabetes, Statins, Smoking, Betablocker, and Erythropoietin the coding was no = 0 and yes = 1. Dots refer to $p<0.05$, while crosses represent p-values below the significance threshold corrected for multiple testing by the Bonferroni method ($\alpha = 0.00017$ in (A), 7.4×10^{-5} in (B), and in (C)).

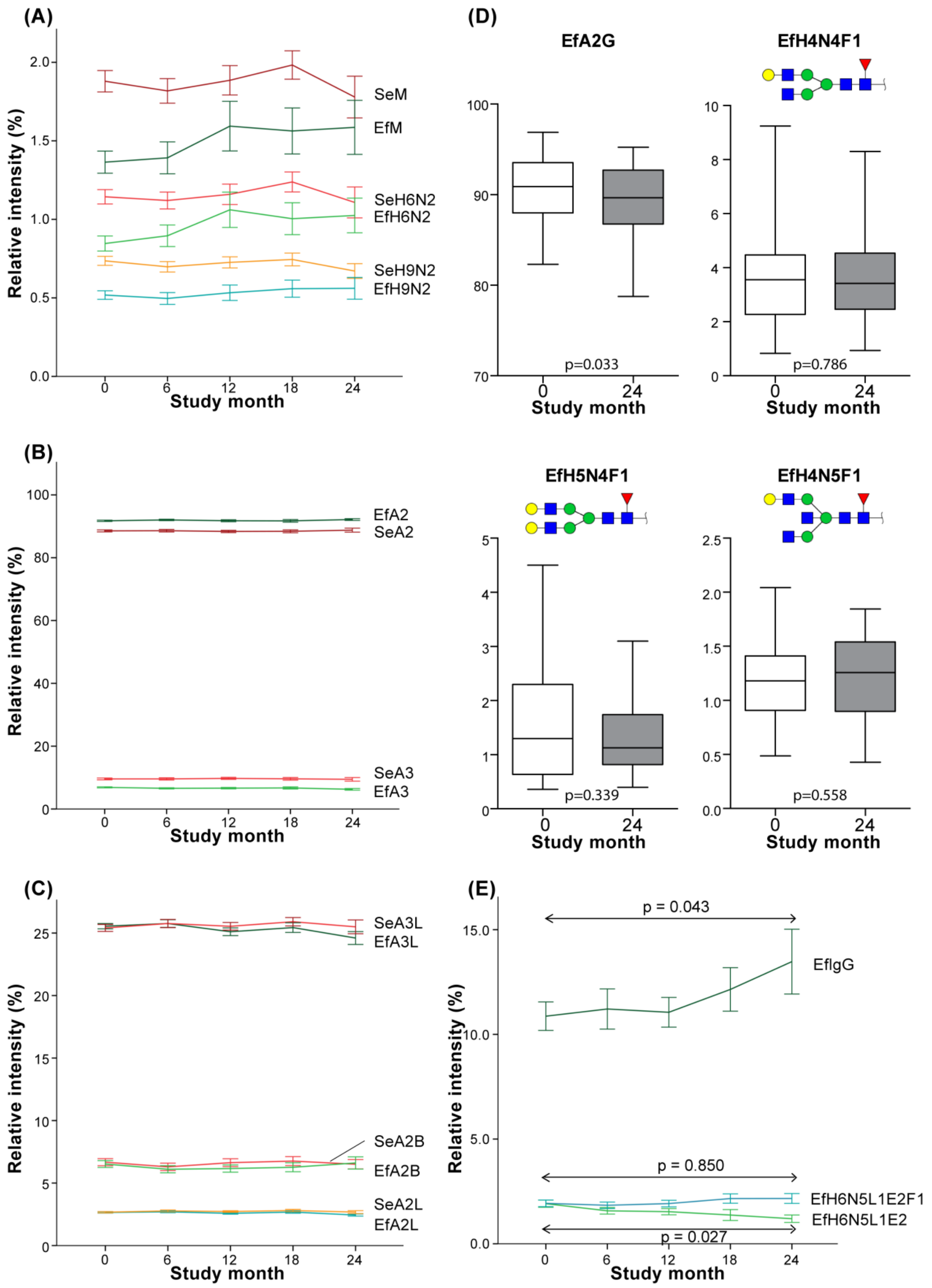

Supporting Information Figure S-3. Relative areas of selected *N*-glycan traits at baseline and over time. A-C) Values of all patients over time classes as defined in Table 1. A) High-mannose type direct traits H6N2 and H9N2 and derived trait M; D) Min/Max-relative-area values and boxplots of 24 patients are shown with p-values from a paired Wilcoxon signed rank test for effluent glycan traits at baseline *vs*. 24 months, *i.e.* galactosylation of diantennary glycans (EfA2G), H4N4F1, H5N4F1, and H4N5F1; E) Values of PD fluid group 2 (Dianeal/Physioneal) over time classes as defined in Table 1. The p-values from a paired Wilcoxon signed-rank test for the relative area of glycan traits at baseline *vs.* 24 months from 12 patients are shown.

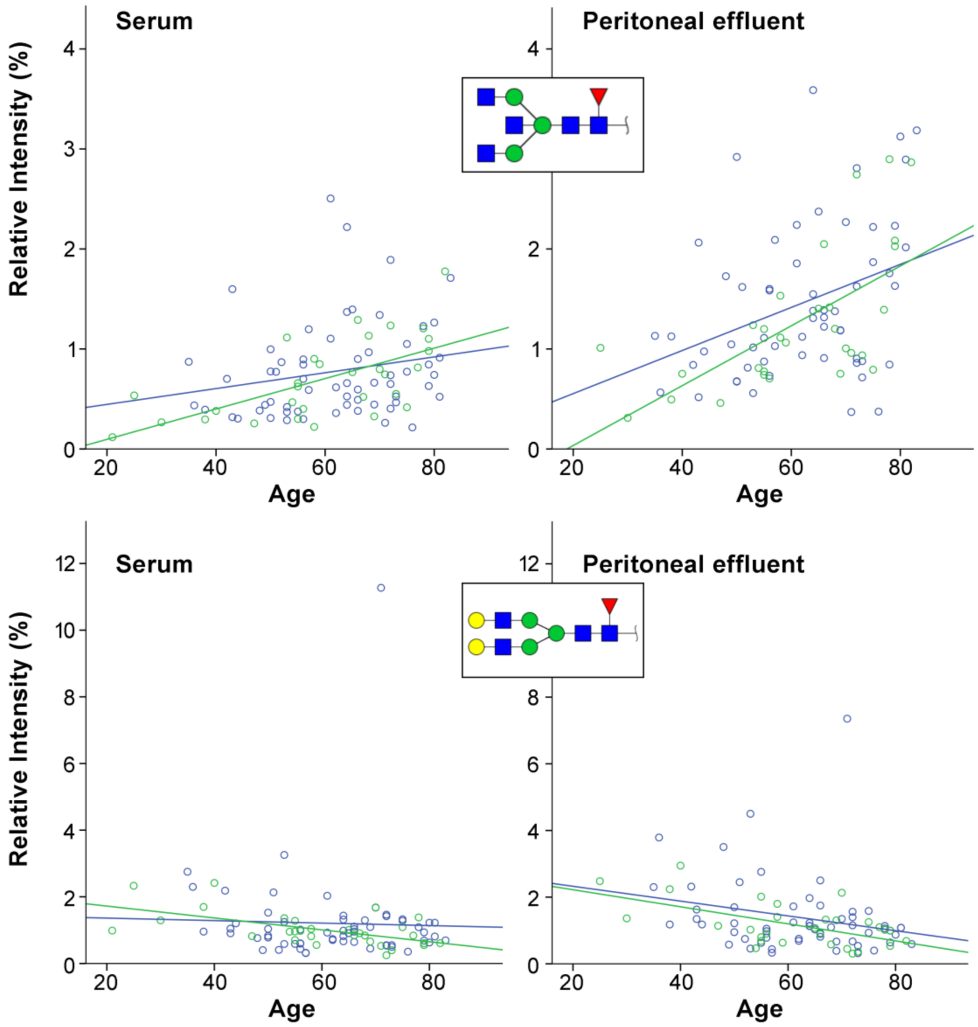

Supporting Information Figure S-4. Age and sex-dependency of *N*-glycans in serum and peritoneal effluent. Blue square, *N*-acetylglucosamine; yellow circle, galactose; green circle, mannose; red triangle, fucose. Pearson's correlation analysis from cross-sectional data is depicted from samples collected at baseline (t=0) from 63 male (blue) and 31 female (green) patients. Upper panel, glycan peak 4: in serum, R^2=0.042 and 0.390 for males and females, respectively, and in peritoneal effluent R^2=0.126 and 0.401 for males and females, respectively. Lower panel, glycan peak 5: in serum, R^2=0.001 and 0.332 for males and females, respectively, and in peritoneal effluent R^2=0.058 and 0.323 for males and females, respectively.

Supporting Information Table S-3A. Associations of peritonitis with glycans according to logistic regression analysis with GEE.

Glycan [a]	Model [b]	Odds ratio (95% CI) [c]	p-value
Ef_A2F	Crude	1.033 (0.984; 1.084)	0.188
	Model 1	1.044 (0.991; 1.099)	0.106
	Model 2	1.036 (0.995; 1.079)	0.086
	Model 1+2	**1.047 (1.000; 1.097)**	**0.048**
Ef_A2G	Crude	0.912 (0.832; 1.000)	0.050
	Model 1	**0.882 (0.788; 0.988)**	**0.030**
	Model 2	**0.899 (0.822; 0.983)**	**0.019**
	Model 1+2	**0.871 (0.784; 0.967)**	**0.010**
Ef_A2E	Crude	0.955 (0.896; 1.018)	0.158
	Model 1	0.941 (0.877; 1.001)	0.088
	Model 2	0.948 (0.897; 1.003)	0.062
	Model 1+2	**0.934 (0.876; 0.996)**	**0.037**
Ef_A3E	Crude	**1.205 (1.004; 1.446)**	**0.046**
	Model 1	1.201 (0.998; 1.444)	0.052
	Model 2	1.191 (0.995; 1.425)	0.057
	Model 1+2	1.191 (0.991; 1.432)	0.063
Ef_IgG	Crude	1.080 (0.993; 1.175)	0.073
	Model 1	**1.105 (1.004; 1.217)**	**0.041**
	Model 2	**1.091 (1.007; 1.181)**	**0.033**
	Model 1+2	**1.116 (1.017; 1.225)**	**0.020**
Ef_H3N4F1	Crude	**1.195 (1.195; 1.377)**	**0.014**
	Model 1	**1.268 (1.061; 1.514)**	**0.009**
	Model 2	**1.226 (1.063; 1.414)**	**0.005**
	Model 1+2	**1.297 (1.098; 1.532)**	**0.002**
Ef_H3N5F1	Crude	**1.651 (1.031; 2.645)**	**0.037**
	Model 1	**2.156 (1.192; 3.898)**	**0.011**
	Model 2	**1.682 (1.075; 2.631)**	**0.023**
	Model 1+2	**2.147 (1.227; 3.754)**	**0.007**

[a] Ef, Effluent; A2F, fucosylation of diantennary glycans; A2G, galactosylation of diantennary glycans; A2E, α2,6-N-acetylneuraminic acids per diantennary glycan; A3E, α2,6-N-acetylneuraminic acids per triantennary glycan; IgG, presumably IgG-related glycans (all diantennary, fucosylated, non-sialylated glycans). Association for direct traits with the compositions H3N4F1 and H3N5F1 was only tested for effluent, but not serum. Associations of serum glycans were not significant ($p>0.05$), and are therefore not shown here for reasons of simplicity.

[b] Model 1: Adjusted for demographics (Age, Sex, Diabetes); Model 2: Adjusted for clinical parameters (continuous vs. automated ambulatory peritoneal dialysis (CAPD/APD), Total Time on PD, Dianeal/Physioneal); Model 1+2: Adjusted for demographics and clinical parameters (Age, Sex, Diabetes, CAPD/APD, Total Time on PD, Dianeal/Physioneal).

[c] Relative signal intensities of the derived glycan traits were calculated as %-values.

Supporting Information Table S-3B. Associations of inflammatory and transport parameters with glycans according to linear regression analysis with GEE.

Glycan[a]	Model[b]	DPCrea4[c]		UF_PET[c]		Ln (TGFβ-1)[c]		Ln (VEGF)[c]		Ln (IL-6)[c]		Ln (IL-8)[c]		Ln (CA-125)[c]		Ln (HA)[c]		Ln (MCP-1)[c]	
		B (95% CI)	p-value	B (95% CI)	p-value	B (95% CI)	p-value	B (95% CI)	p-value	B (95% CI)	p-value	B (95% CI)	p-value	B (95% CI)	p-value	B (95% CI)	p-value	B (95% CI)	p-value
SeA2	Crude	**-0.005 (-0.010; 0.0004)**	**0.032**	-10.408 (-27.324; 6507)	0.228	**-0.037 (-0.071; -0.002)**	**0.036**	**-0.089 (-0.141; -0.037)**	**0.0010**	**-0.075 (-0.120; -0.031)**	**0.001**	**-0.069 (-0.125; -0.012)**	**0.017**	-0.003 (-0.035; 0.028)	0.829	-0.018 (-0.054; 0.019)	0.342	**-0.038 (-0.075; -0.001)**	**0.044**
	Model 1	-0.004 (-0.009; 0.0003)	0.070	-9.984 (-26.649; 6.681)	0.240	-0.034 (-0.067; 0.0002)	0.051	**-0.088 (-0.139; -0.037)**	**0.0010**	**-0.065 (-0.108; -0.022)**	**0.003**	**-0.061 (-0.114; -0.008)**	**0.024**	-0.002 (-0.033; 0.029)	0.901	-0.016 (-0.052; 0.019)	0.375	-0.033 (-0.066; -0.001)	0.054
	Model 2	**-0.005 (-0.010; -0.001)**	**0.024**	-9.665 (-26.834; 7.505)	0.270	**-0.040 (-0.073; -0.007)**	**0.017**	**-0.094 (-0.146; -0.042)**	**0.0004**	**-0.078 (-0.122; -0.033)**	**0.001**	**-0.061 (-0.114; -0.008)**	**0.024**	-0.009 (-0.038; 0.020)	0.541	-0.018 (-0.054; 0.019)	0.345	**-0.039 (-0.075; -0.004)**	**0.031**
	Model 1+2	-0.005 (-0.009; 0.0002)	0.059	-8.736 (-26.320; 8.849)	0.330	**-0.038 (-0.071; -0.005)**	**0.024**	**-0.093 (-0.144; -0.042)**	**0.0003**	**-0.071 (-0.116; -0.026)**	**0.002**	**-0.060 (-0.111; -0.009)**	**0.021**	-0.009 (-0.038; 0.019)	0.522	-0.016 (-0.053; 0.020)	0.377	**-0.035 (-0.068; -0.002)**	**0.039**
SeA3	Crude	**0.005 (0.0004; 0.010)**	**0.033**	15215 (-3.700; 34.130)	0.115	**0.039 (0.003; 0.076)**	**0.035**	**0.093 (0.038; 0.148)**	**0.001**	**0.077 (0.028; 0.125)**	**0.002**	**0.073 (0.011; 0.136)**	**0.021**	0.002 (-0.031; 0.035)	0.971	0.014 (-0.024; 0.051)	0.479	0.033 (-0.006; 0.072)	0.102
	Model 1	0.005 (0.000; 0.010)	0.065	14.708 (3.77; 33.187)	0.119	**0.037 (0.001; 0.073)**	**0.046**	**0.093 (0.039; 0.148)**	**0.001**	**0.069 (0.022; 0.116)**	**0.004**	**0.067 (0.009; 0.126)**	**0.024**	-0.00005 (-0.033; 0.033)	0.998	0.013 (-0.024; 0.050)	0.493	0.029 (-0.006; 0.065)	0.108
	Model 2	**0.006 (0.001; 0.010)**	**0.022**	14.635 (- 4.305; 33.575)	0.130	**0.043 (0.008; 0.078)**	**0.017**	**0.098 (0.042; 0.153)**	**0.001**	**0.079 (0.031; 0.128)**	**0.001**	**0.072 (0.010; 0.134)**	**0.023**	0.007 (-0.024; 0.037)	0.675	0.014 (-0.024; 0.052)	0.474	0.035 (-0.003; 0.072)	0.074
	Model 1+2	0.005 (-0.00001; 0.010)	0.050	13.558 (-5.632; 32.749)	0.166	**0.041 (0.006;0.076)**	**0.021**	**0.098 (0.044; 0.152)**	**0.0004**	**0.074 (0.027; 0.122)**	**0.002**	**0.067 (0.010; 0.124)**	**0.021**	0.006 (-0.024; 0.037)	0.676	0.013 (-0.024; 0.051)	0.485	0.031 (-0.004; 0.067)	0.081
SeA3F	Crude	0.001 (-0.001; 0.002)	0.302	-0.246 (-4.420; 3.928)	0.908	-0.0006 (-0.009; 0.008)	0.989	0.006 (-0.006; 0.017)	0.348	**0.014 (0.001; 0.026)**	**0.031**	**0.020 (0.004; 0.035)**	**0.012**	-0.001 (-0.009; 0.007)	0.782	0.004 (-0.003; 0.011)	0.231	0.007 (-0.002; 0.016)	0.113
	Model 1	0.0005 (-0.001; 0.002)	0.427	0.8850 (-3.064; 4.763)	0.670	-0.001 (-0.010; 0.007)	0.772	0.003 (-0.008; 0.014)	0.606	0.012 (-0.001; 0.024)	0.063	**0.016 (0.000; 0.032)**	**0.044**	0.00004 (-0.009; 0.009)	0.992	0.003 (-0.004; 0.010)	0.409	0.004 (-0.004; 0.013)	0.290
	Model 2	0.001 (-0.001; 0.002)	0.266	0.824 (-3.081; 4.678)	0.675	0.001 (-0.008; 0.009)	0.896	0.005 (-0.006; 0.017)	0.375	0.011 (-0.002; 0.025)	0.089	**0.019 (0.003; 0.036)**	**0.019**	-0.001 (-0.007; 0.010)	0.727	0.003 (-0.004; 0.010)	0.346	0.006 (-0.003; 0.015)	0.169
	Model 1+2	0.001 (-0.001; 0.002)	0.381	1.93 (-1.686; 5.554)	0.295	0.0005 (-0.009; 0.008)	0.909	0.004 (-0.007; 0.015)	0.527	0.009 (-0.004; 0.021)	0.166	**0.015 (-0.001; 0.032)**	**0.062**	0.002 (-0.007; 0.011)	0.648	0.002 (-0.005; 0.010)	0.548	0.004 (-0.004; 0.012)	0.361
SeA2B	Crude	0.004 (-0.001; 0.010)	0.122	-3.587 (-25.844; 18.670)	0.752	-0.009 (-0.056; 0.038)	0.699	0.032 (-0.033; 0.097)	0.332	0.031 (-0.031; 0.094)	0.331	**0.095 (0.025; 0.164)**	**0.008**	-0.029 (-0.075; 0.017)	0.223	0.027 (-0.017; 0.071)	0.232	0.018 (-0.032; 0.067)	0.482
	Model 1	0.004 (-0.002; 0.009)	0.216	1.134 (-20.292; 22.560)	0.917	-0.019 (-0.068; 0.028)	0.420	0.019 (-0.048; 0.085	0.583	0.004 (-0.061; 0.069)	0.902	**0.062 (-0.008; 0.132)**	**0.082**	-0.027 (-0.072; 0.018)	0.241	0.019 (-0.028; 0.066)	0.422	0.001 (-0.045; 0.047)	0.957
	Model 2	0.004 (-0.001; 0.009)	0.138	-1.972 (-22.701; 18.757)	0.852	-0.002 (-0.046; 0.049)	0.950	0.033 (-0.033; 0.100)	0.326	0.029 (-0.030; 0.088)	0.341	**0.098 (0.029; 0.167)**	**0.005**	-0.014 (-0.054; 0.026)	0.502	0.026 (-0.018; 0.070)	0.247	0.014 (-0.030; 0.059)	0.533
	Model 1+2	0.003 (-0.002; 0.009)	0.276	1.889 (-18.334; 22.112)	0.855	-0.007 (-0.056; 0.042)	0.789	0.023 (-0.045; 0.092)	0.509	0.006 (-0.057; 0.070)	0.842	**0.068 (0.001; 0.135)**	**0.048**	-0.013 (-0.053; 0.028)	0.541	0.019 (-0.027; 0.066)	0.418	0.0002 (-0.043; 0.043)	0.993
SeA2G	Crude	-0.003 (-0.007; 0.002)	0.253	-3.253 (-25.318; 18.812)	0.773	-0.019 (-0.043; 0.005)	0.128	-0.013 (-0.042; 0.016)	0.228	-0.012 (-0.049; 0.025)	0.531	-0.054 (-0.104; -0.003)	0.038	0. 019 (-0.004; 0.042)	0.111	-0.010 (-0.031; 0.010)	0.323	-0. 012 (-0.038; 0.014)	0.367
	Model 1	-0.003 (-0.008; 0.002)	0.213	-1.492 (-20.937; 17.957)	0.880	-0.017 (-0.044; 0.011)	0.229	-0.004 (-0.038; 0.030)	0.811	0.010 (-0.027; 0.046)	0.599	-0.032 (-0.083; 0.018)	0.211	0. 019 (-0.006; 0.045)	0.141	-0.002 (-0.027; 0.022)	0.848	0.0005 (-0.027; 0.028)	0.974
	Model 2	-0.003 (-0.008; 0.001)	0.190	-1.062 (-22.713; 20.589)	0.923	**-0.028 (-0.053; -0.004)**	**0.025**	-0.017 (-0.046; 0.012)	0.243	-0.008 (-0.046; 0.031)	0.698	-0.063 (-0.116; 0.009)	0.022	0. 015 (-0.010; 0.040)	0.232	-0.010 (-0.030; 0.010)	0.329	-0. 007 (-0.034; 0.020)	0.615
	Model 1+2	-0.003 (-0.008; 0.002)	0.230	2.625 (-16.778; 22.027)	0.791	-0.026 (-0.054; 0.002)	0.068	-0.006 (-0.40; 0.028)	0.721	0.013 (-0.025; 0.051)	0.514	-0.038 (-0.092; 0.015)	0.161	0. 014 (-0.013; 0.041)	0.301	-0.003 (-0.026; 0.021)	0.821	0.004 (-0.023; 0.032)	0.749
SeA3E	Crude	-0.00003 (-0.006; 0.006)	0.993	-3.253 (-25.318; 18.812)	0.773	0.018 (-0.020; 0.055)	0.354	0.033 (-0.034; 0.100)	0.336	**0.088 (0.020; 0.157)**	**0.011**	0.040 (-0.031; 0.111)	0.267	0.004 (-0.039; 0.048)	0.839	0.024 (-0.024; 0.072)	0.335	0.010 (-0.030; 0.050)	0.625
	Model 1	0.0001 (-0.006; 0.006)	0.964	-1.492 (-20.937; 17.954)	0.880	0.017 (-0.020; 0.054)	0.364	0.033 (-0.034; 0.101)	0.333	**0.088 (0.024; 0.153)**	**0.007**	0.030 (-0.041; 0.101)	0.402	0.003 (-0.039; 0.045)	0.833	0.021 (-0.027; 0.069)	0.387	0.009 (-0.027; 0.046)	0.616
	Model 2	-0.0002 (-0.007; 0.007)	0.956	-1.062 (-22.713; 20.589)	0.923	0.028 (-0.007; 0.063)	0.111	0.038 (-0.032; 0.107)	0.288	**0.093 (0.025; 0.160)**	**0.007**	0.035 (-0.037; 0.106)	0.341	0.011 (-0.033; 0.055)	0.619	0.024 (-0.027; 0.074)	0.354	0.018 (-0.021; 0.056)	0.376
	Model 1+2	-0.0003 (-0.007; 0.006)	0.936	2625 (-16.678; 22.027)	0.791	0.026 (-0.008; 0.060)	0.134	0.033 (-0.039; 0.104)	0.370	**0.088 (0.023; 0.152)**	**0.007**	0.014 (-0.057; 0.084)	0.707	0.013 (-0.031; 0.056)	0.565	0.020 (-0.031; 0.071)	0.446	0.014 (-0.023; 0.052)	0.455
EfA2	Crude	**-0.008 (-0.016; -0.001)**	**0.019**	-13.602 (-37.791; 10.588)	0.270	**-0.055 (-0.091; -0.020)**	**0.002**	-0.060 (-0.127; 0.007)	0.077	-0.025 (-0.099; 0.049)	0.509	0.086 (-0.175; 0.004)	0.061	-0.012 (-0.078; 0.054)	0.725	-0.022 (-0.064; 0.019)	0.292	-0.024 (-0.070; 0.021)	0.297
	Model 1	**-0.008 (-0.015; -0.0004)**	**0.040**	-12.029 (-37.417; 13.359)	0.353	**-0.053 (-0.088; -0.017)**	**0.003**	-0.058 (-0.126; 0.009)	0.090	-0.016 (-0.091; 0.058)	0.667	-0.073 (-0.159; 0.013)	0.098	-0.012 (-0.078; 0.054)	0.717	-0.020 (-0.062; 0.021)	0.335	-0.019 (-0.064; 0.026)	0.408
	Model 2	**-0.009 (-0.017; -0.002)**	**0.015**	-12.178 (-37.235; 12.879)	0.341	**-0.059 (-0.100; -0.019)**	**0.004**	-0.073 (-0.148; 0.002)	0.057	-0.048 (-0.131; -0.035)	0.260	**-0.107 (-0.203; -0.011)**	**0.029**	-0.001 (-0.074; 0.071)	0.972	-0.028 (-0.076; 0.019)	0.244	-0.025 (-0.081; 0.030)	0.372
	Model 1+2	**-0.009 (-0.016; -0.001)**	**0.026**	-8.825 (-34.912; 17.272)	0.507	**-0.057 (-0.098; -0.017)**	**0.006**	-0.073 (-0.149; 0.002)	0.058	-0.043 (-0.126; 0.040)	0.310	**-0.101 (-0.190; -0.013)**	**0.024**	-0.002 (-0.077; 0.072)	0.951	-0.027 (-0.074; 0.020)	0.258	-0.023 (-0.078; 0.032)	0.417
EfA3	Crude	**0.012 (0.003; 0.020)**	**0.007**	21.536 (-7.036; 50.108)	0.140	**0.083 (0.037; 0.129)**	**0.0004**	**0.108 (0.017; 0.200)**	**0.020**	0.085 (-0.008; 0.178)	0.073	**0.750 (0.019; 0.130)**	**0.008**	0.043 (-0.023; 0.109)	0.203	0.045 (-0.015; 0.105)	0.142	-0.053 (-0.001; 0.108)	0.057
	Model 1	**0.011 (0.002; 0.020)**	**0.013**	19.640 (-11.620; 50.399)	0.211	**0.080 (0.033; 0.126)**	**0.0008**	**0.107 (0.013; 0.200)**	**0.026**	0.076 (-0.020; 0.172)	0.122	0.073 (-0.159; 0.013)	0.098	0.045 (-0.022; 0.112)	0.185	0.042 (-0.018; 0.103)	0.169	0.048 (-0.007; 0.102)	0.089
	Model 2	**0.013 (0.004; 0.022)**	**0.003**	18.224 (- 9.593; 46.042)	0.199	**0.089 (0.040; 0.137)**	**0.0003**	**0.123 (0.028; 0.217)**	**0.011**	**0.121 (0.032; 0.210)**	**0.007**	**0.107 (0.203; 0.011)**	**0.029**	0.032 (-0.038; 0.102)	0.367	0.054 (-0.011; 0.120)	0.106	0.057 (-0.005; 0.119)	0.072
	Model 1+2	**0.013 (0.004; 0.022)**	**0.004**	13.832 (-15.947; 43.610)	0.363	**0.088 (0.039; 0.137)**	**0.0004**	**0.125 (0.029; 0.220)**	**0.011**	**0.117 (0.027; 0.208)**	**0.007**	**0.140 (0.029; 0.252)**	**0.014**	0.035 (-0.036; 0.107)	0.334	0.054 (-0.012; 0.119)	0.110	0.055 (-0.007; 0.116)	0.080
EfA2F	Crude	-0.0001 (-0.002; 0.002)	0.929	0.973 (-6.409; 8.355)	0.796	-0.006 (-0.018; 0.007)	0.361	-0.008 (-0.029; 0.013)	0.459	0.001 (-0.021; 0.024)	0.901	0.016(-0.009; 0.041)	0.222	**-0.022 (-0.042; -0.002)**	**0.028**	-0.001 (-0.012; 0.010)	0.893	-0.001 (-0.015; 0.013)	0.880
	Model 1	-0.0004 (-0.003; 0.002)	0.709	3.627 (-3.276; 10.530)	0.303	-0.008 (-0.021; 0.005)	0.229	-0.013 (-0.035; 0.008)	0.232	-0.008 (-0.032; 0.017)	0.545	0.002 (-0.022; 0.027)	0.851	**-0.022 (-0.043; -0.002)**	**0.033**	-0.004 (-0.016; 0.007)	0.447	-0.008 (-0.021; 0.006)	0.249
	Model 2	-0.0001 (-0.002; 0.002)	0.905	2.401 (-4.862; 9.66)	0.517	-0.005 (-0.018; 0.007)	0.418	-0.008 (-0.029; 0.013)	0.453	0.001 (-0.022; 0.023)	0.956	0.020 (-0.005; 0.045)	0.111	**-0.023 (-0.039; -0.006)**	**0.008**	-0.001 (-0.012; 0.010)	0.838	-0.004 (-0.018; 0.010)	0.560
	Model 1+2	-0.001 (-0.003; 0.002)	0.613	4.103 (-2.673; 10.880)	0.235	-0.007; (-0.020; 0.006)	0.291	-0.013 (-0.035; 0.008)	0.228	-0.007 (-0.031; 0.017)	0.560	0.007 (-0.017; 0.031)	0.576	**-0.023 (-0.041; -0.005)**	**0.013**	-0.004 (-0.016; 0.007)	0.459	-0.010 (-0.022; 0.003)	0.144
EfA3F	Crude	0.001 (0.000; 0.002)	0.181	-1.237 (-5.280; 2.806)	0.549	0.004 (-0.005; 0.012)	0.376	0.011 (-0.002; 0.024)	0.088	**0.026 (0.012; 0.040)**	**0.0003**	0.018 (0.004; 0.033)	0.015	0.004 (-0.006; 0.015)	0.394	0.006 (-0.001; 0.014)	0.115	**0.010 (0.001; 0.019)**	**0.037**
	Model 1	0.001 (-0.001; 0.002)	0.323	-0.660 (-4.401; 3.082)	0.730	0.003 (-0.006; 0.012)	0.503	0.010 (-0.003; 0.023)	0.136	**0.025 (0.010; 0.039)**	**0.0007**	0.014 (-0.001; 0.028)	0.059	0.005 (-0.005; 0.015)	0.359	0.005 (-0.003; 0.013)	0.198	0.008 (-0.001; 0.017)	0.084
	Model 2	0.001 (0.000; 0.002)	0.136	-0.043 (-3.747; 3.661)	0.982	0.004 (-0.005; 0.012)	0.378	0.012 (-0.001; 0.025)	0.061	**0.026 (0.012; 0.040)**	**0.0003**	0.017 (0.003; 0.032)	0.015	0.006 (-0.003; 0.015)	0.196	0.005 (-0.002; 0.013)	0.151	**0.010 (0.001; 0.019)**	**0.030**
	Model 1+2	0.001 (0.000; 0.002)	0.246	0.568 (-2.818; 3.958)	0.742	0.003 (-0.005; 0.011)	0.498	0.011 (-0.002; 0.024)	0.098	**0.024 (0.010; 0.038)**	**0.0009**	0.013 (-0.001; 0.026)	0.060	0.007 (-0.003; 0.016)	0.167	0.004 (-0.003; 0.012)	0.276	0.008 (-0.001; 0.017)	0.075
EfA2B	Crude	0.002 (-0.004; 0.008)	0.511	4.279 (-21.002; 29.560)	0.740	-0.003 (-0.041; 0.035)	0.889	-0.015 (-0.077; 0.047)	0.629	0.043 (-0.020; 0.106)	0.181	**0.123 (0.045; 0.201)**	**0.002**	**-0.053 (-0.104; -0.001)**	**0.045**	0.028 (-0.005; 0.061)	0.099	-0.002 (-0.050; 0.045)	0.928
	Model 1	0.0004 (-0.006; 0.007)	0.911	11.440 (-12.763; 35.643)	0.354	-0.012 (-0.051; .0.026)	0.531	-0.035 (-0.096; 0.027)	0.270	0.011 (-0.065; 0.087)	0.775	0.071 (-0.003; 0.145)	0.059	**-0.052 (-0.104; -0.0001)**	**0.049**	0.017 (-0.019; 0.053)	0.361	-0.029 (-0.069; 0.012)	0.163
	Model 2	0.002 (-0.004; 0.008)	0.516	8.675 (-16.675; 34.026)	0.502	-0.011 (-0.024; 0.045)	0.550	-0.011 (-0.074; 0.051)	0.721	0.046 (-0.019; 0.111)	0.166	**0.132 (0.053; 0.211)**	**0.001**	-0.040 (-0.086; -0.005)	0.080	0.024 (-0.009; 0.058)	0.152	-0.004 (-0.047; 0.040)	0.869
	Model 1+2	-0.00007 (-0.007; 0.006)	0.982	13.010 (-11.087; 37.105)	0.290	0.003 (-0.034; 0.040)	0.870	-0.030 (-0.093; 0.033)	0.354	0.017 (-0.060; 0.095)	0.663	**0.076 (0.002; 0.151)**	**0.045**	-0.039 (-0.086; 0.008)	0.103	0.014 (-0.023; 0.051)	0.447	-0.026 (-0.066; 0.013)	0.188
EfA2G	Crude	-0.002 (-0.006; 0.002)	0.264	4.815 (-7.713; 17.345)	0.451	-0.005 (-0.030; 0.020)	0.714	0.012 (-0.027; 0.051)	0.549	-0.016 (-0.055; 0.023)	0.427	**-0.053 (-0.094; -0.011)**	**0.013**	**0.052 (0.021; 0.084)**	**0.001**	-0.006 (-0.028; 0.016)	0.606	-0.007 (-0.031; 0.016)	0.532
	Model 1	-0.002 (-0.006; 0.002)	0.335	-2.061 (-15.598; 11.475)	0.765	-0.002 (-0.029; 0.026)	0.895	0.025 (-0.019; 0.068)	0.262	0.007 (-0.036; 0.050)	0.738	-0.024 (-0.70; 0.021)	0.291	**0.056 (0.022; 0.090)**	**0.001**	0.002 (-0.022; 0.027)	0.847	0.007 (-0.019; 0.032)	0.612
	Model 2	-0.002 (-0.006; 0.002)	0.260	-0.037 (-12.465; 11.728)	0.952	-0.013 (-0.038; 0.012)	0.296	0.010 (-0.029; 0.048)	0.621	-0.010 (-0.049; 0.029)	0.622	**-0.059 (-0.103; -0.016)**	**0.008**	**0.045 (0.016; 0.074)**	**0.002**	-0.003 (-0.026; 0.019)	0.787	-0.001 (-0.024; 0.023)	0.946
	Model 1+2	-0.002 (-0.006; 0.003)	0.477	-6.151 (-18.711; 6.410)	0.337	-0.012 (-0.040; 0.016)	0.399	0.023 (-0.020; 0.066)	0.299	0.011 (-0.031; 0.053)	0.602	-0.028 (-0.075; 0.019)	0.247	**0.048 (0.016; 0.080)**	**0.004**	0.004 (-0.020; 0.029)	0.734	0.012 (-0.013; 0.037)	0.346
EfA2L	Crude	**-0.030 (-0.058; -0.01)**	**0.041**	36.943 (-71.567; 145.453)	0.505	-0.126 (-0.295; 0.042)	0.142	-0.124 (-0.383; 0.134)	0.346	**-0.487 (-0.780; -0.194)**	**0.001**	-0.053 (-0.403; 0.297)	0.766	-0.068 (-0.291; 0.154)	0.547	-0.092 (-0.271; 0.087)	0.315	-0.106 (-0.288; 0.077)	0.257
	Model 1	**-0.034 (-0.064; -0.004)**	**0.025**	19.166 (-83.988; 122.320)	0.716	-0.132 (-0.308; 0.044)	0.141	-0.107 (-0.370; 0.156)	0.425	**-0.444 (-0.747; -0.140)**	**0.004**	0.057(-0.268; 0.383)	0.729	-0.078 (-0.301; 0.146)	0.496	-0.070 (-0.255; 0.115)	0.460	-0.074 (-0.255; 0.107)	0.423
	Model 2	**-0.030 (-0.058; -0.001)**	**0.042**	-3.298 (-118.573; 111.977)	0.955	-0.161 (-0.330; 0.009)	0.063	-0.154 (-0.419; 0.110)	0.253	**-0.455 (-0.762; -0.149)**	**0.007**	-0.009 (-0.374; -0.356)	0.962	-0.107 (-0.338; 0.125)	0.366	-0.083 (-0.272; 0.105)	0.385	-0.093 (-0.275; 0.089)	0.317
	Model 1+2	**-0.032 (-0.061; -0.002)**	**0.036**	-23.567 (-129.805; 82.671)	0.664	-0.170 (-0.346; 0.005)	0.058	-0.123 (-0.396; 0.150)	0.377	**-0.403 (-0.723; -0.082)**	**0.014**	0.156 (-0.163; 0.475)	0.338	-0.131 (-0.370; 0.108)	0.284	-0.061 (-0.258; 0.137)	0.547	-0.058 (-0.244; 0.128)	0.543
EfA3L	Crude	-0.003 (-0.009; 0.004)	0.401	18.954 (-2.912; 40.819)	0.089	0.0004 (-0.038; 0.037)	0.982	-0.001 (-0.067; 0.066)	0.983	-0.001 (-0.079; 0.076)	0.975	0.054 (-0.017; 0.126)	0.136	0.042 (-0.011; 0.095)	0.122	-0.006 (-0.050; 0.038)	0.779	0.021 (-0.025; 0.066)	0.371
	Model 1	-0.003 (-0.009; 0.003)	0.315	16.302 (-4.960; 37.563)	0.133	-0.002 (-0.042; 0.037)	0.913	0.002 (-0.063; 0.067)	0.954	0.010 (-0.066; 0.086)	0.797	0.062 (-0.001; 0.126)	0.054	0.042 (-0.011; 0.095)	0.121	-0.004 (-0.046; 0.039)	0.862	0.026 (-0.015; 0.066)	0.211
	Model 2	-0.002 (-0.009; 0.005)	0.572	8.885 (-14.643; 32.413)	0.519	-0.001 (-0.039; 0.036)	0.941	-0.005 (-0.073; 0.064)	0.895	0.016 (-0.067; 0.099)	0.709	0.067 (-0.007; 0.140)	0.075	0.038 (-0.016; 0.092)	0.171	-0.001 (-0.047; 0.045)	0.964	0.029 (-0.019; 0.078)	0.234
	Model 1+2	-0.002 (-0.009; 0.004)	0.479	5.518 (-17.525; 28.562)	0.639	-0.003 (-0.041; 0.035)	0.883	-0.0002 (-0.067; 0.067)	0.996	0.025 (-0.055; 0.105)	0.538	**0.080 (0.015; 0.144)**	**0.016**	0.038 (-0.018; 0.093)	0.187	0.002 (-0.043; 0.046)	0.944	0.034 (-0.010; 0.078)	0.126
EfA2E	Crude	0.00003 (-0.003; 0.003)	0.986	-2.117 (-11.341; 7.106)	0.653	0.008 (-0.008; 0.024)	0.327	0.015 (-0.014; 0.044)	0.304	0.007 (-0.024; 0.039)	0.643	-0.023 (-0.055; 0.008)	0.149	**0.033 (0.009; 0.056)**	**0.007**	0.002 (-0.014; 0.018)	0.824	0.002 (-0.016; 0.020)	0.826
	Model 1	0.0002 (-0.003; 0.003)	0.873	-5.313 (-14.226; 3.600)	0.243	0.010 (-0.007; 0.027)	0.248	0.021 (-0.008; 0.050)	0.165	0.018 (-0.014; 0.050)	0.274	-0.011 (-0.043; 0.021)	0.502	**0.033 (0.008; 0.057)**	**0.008**	0.006 (-0.010; 0.022)	0.480	0.009 (-0.008; 0.026)	0.306
	Model 2	0.00003 (-0.003; 0.003)	0.985	-3.676 (-12.594; 5.241)	0.419	0.006 (-0.011; 0.023)	0.487	0.014 (-0.014; 0.043)	0.326	0.008 (-0.022; 0.039)	0.596	-0.030 (-0.062; 0.002)	0.063	**0.032 (0.011; 0.052)**	**0.002**	0.003 (-0.013; 0.019)	0.740	0.006 (-0.012; 0.023)	0.537
	Model 1+2	0.0004 (0.003; 0.003)	0.779	-5.589 (-14.187; 3.008)	0.203	0.008 (-0.010; 0.025)	0.398	0.020 (-0.009; 0.049)	0.184	0.017 (-0.015; 0.048)	0.293	-0.018 (-0.049; 0.014)	0.271	**0.032 (0.010; 0.054)**	**0.004**	0.006 (-0.010; 0.022)	0.461	0.011 (-0.006; 0.028)	0.202
EfA3E	Crude	**0.005 (0.001; 0.009)**	**0.019**	-3.981 (-18.568; 10.606)	0.159	**0.046 (0.021; 0.071)**	**0.0003**	**0.082 (0.044; 0.121)**	**0.00003**	**0.113 (0.057; 0.169)**	**0.0001**	**0.075 (0.019; 0.130)**	**0.008**	0.036 (-0.006; 0.079)	0.096	**0.049 (0.023; 0.076)**	**0.0003**	**0.041 (0.012; 0.070)**	**0.005**
	Model 1	**0.005 (0.001; 0.010)**	**0.013**	-4.040 (-18.391; 10.312)	0.581	**0.047 (0.021; 0.074)**	**0.0005**	**0.083 (0.044; 0.122)**	**0.00003**	**0.109 (0.054; 0.165)**	**0.0001**	**0.062 (0.008; 0.117)**	**0.026**	0.038 (-0.005; 0.082)	0.084	**0.049 (0.023; 0.075)**	**0.0002**	**0.040 (0.012; 0.068)**	**0.006**
	Model 2	**0.005 (0.001; 0.010)**	**0.016**	-1.528 (-16.128; 13.072)	0.837	**0.049 (0.024; 0.075)**	**0.0002**	**0.087 (0.046; 0.127)**	**0.00003**	**0.115 (0.054; 0.175)**	**0.0002**	**0.073 (0.016; 0.130)**	**0.012**	0.039 (-0.005; 0.084)	0.082	**0.052 (0.025; 0.079)**	**0.0001**	**0.044 (0.013; 0.075)**	**0.005**
	Model 1+2	**0.006 (0.001; 0.010)**	**0.013**	-1.155 (-15.689; 13.378)	0.876	**0.052 (0.026; 0.079)**	**0.0001**	**0.086 (0.045; 0.128)**	**0.00005**	**0.111 (0.051; 0.171)**	**0.0003**	**0.056 (0.001; 0.112)**	**0.045**	0.043 (-0.002; 0.088)	0.059	**0.052 (0.026; 0.079)**	**0.0001**	**0.042 (0.011; 0.072)**	**0.007**
EfIgG	Crude	0.001 (-0.003; 0.005)	0.605	-0.543 (-12.340; 11.254)	0.928	-0.006 (-0.028; 0.015)	0.565	-0.018 (-0.057; 0.020)	0.348	0.007 (-0.034; 0.048)	0.742	0.029 (-0.013; 0.071)	0.174	**-0.045 (-0.072; -0.018)**	**0.001**	-0.001 (-0.022; 0.020)	0.929	0.003 (-0.021; 0.026)	0.832
	Model 1	0.001 (-0.003; 0.005)	0.645	4.737 (-7.250; 16.724)	0.439	-0.009 (-0.032; 0.014)	0.459	-0.027 (-0.068; 0.013)	0.190	-0.008 (-0.051; 0.036)	0.738	0.010 (-0.032; 0.053)	0.634	**-0.046 (-0.075; -0.018)**	**0.001**	-0.007 (-0.029; 0.015)	0.541	-0.007 (-0.030; 0.016)	0.563
	Model 2	0.001 (-0.003; 0.005)	0.599	2.788 (-8.314; 13.890)	0.623	-0.001 (-0.023; 0.021)	0.941	-0.017 (-0.056; 0.022)	0.385	0.002 (-0.039; 0.043)	0.909	0.035 (-0.008; 0.078)	0.116	**-0.041 (-0.066; -0.016)**	**0.001**	-0.003 (-0.024; 0.019)	0.802	-0.002 (-0.025; 0.020)	0.850
	Model 1+2	0.001 (-0.003; 0.004)	0.784	6.753 (-4.121; 17.627)	0.224	-0.003 (-0.026; 0.021)	0.819	-0.026 (-0.066; 0.015)	0.210	-0.010 (-0.053; 0.032)	0.632	0.014 (-0.028; 0.057)	0.508	**-0.042 (-0.069; -0.015)**	**0.002**	-0.008 (-0.030; 0.014)	0.483	-0.010 (-0.032; 0.012)	0.374

[a] Se, Serum; Ef, Effluent; A2, diantennary glycans; A3, triantennary glycans; A3F, fucosylation of triantennary glycans; A2B, bisection of diantennary glycans; A2G, galactosylation of diantennary glycans; A3E, α2,6-N-acetylneuraminic acids per triantennary glycan; A2F, fucosylation of diantennary glycans; A2L, α2,3-N-acetylneuraminic acids per diantennary glycan; A3L, α2,3-N-acetylneuraminic acids per triantennary glycan; A2E, α2,6-N-acetylneuraminic acids per diantennary glycan; IgG, presumably IgG-related glycans (all diantennary, fucosylated, non-sialylated glycans). High-mannose glycans (M) both in serum and effluent, as well as serum glycans A2F, A2L, A3L, A2E, and IgG were not significantly associated with any of the tested clinical parameters and are, therefore, not shown here.

[b] Model 1: Adjusted for demographics (Age, Sex, Diabetes); Model 2: Adjusted for clinical parameters (continuous vs. automated ambulatory peritoneal dialysis (CAPD/APD), Total Time on PD, Dianeal/Physioneal); Model 1+2: Adjusted for demographics and clinical parameters (Age, Sex, Diabetes, CAPD/APD, Total Time on PD, Dianeal/Physioneal)

[c] DPCrea4, Ratio of creatinine in the effluent vs. plasma during 4 h dwell time; UF_PET, Ultrafiltration during PET; TGFβ-1, transforming growth factor-β1; VEGF, vascular endothelial growth factor; IL, interleukin; CA-125, Cancer Antigen 125; HA, hyaluronic acid; MCP-1, monocytes chemotactic protein 1; IL-6, IL-8, MCP-1, HA, TGFβ-1, VEGF and CA-125 were ln-transformed prior to GEE analysis; relative signal intensities of the derived glycan traits were calculated as %-values
